# Characterization of the RNA-dependent RNA polymerase from Chikungunya virus and discovery of a novel ligand as a potential drug candidate

**DOI:** 10.1038/s41598-022-14790-x

**Published:** 2022-06-22

**Authors:** Marjorie C. L. C. Freire, Luis G. M. Basso, Luis F. S. Mendes, Nathalya C. M. R. Mesquita, Melina Mottin, Rafaela S. Fernandes, Lucca R. Policastro, Andre S. Godoy, Igor A. Santos, Uriel E. A. Ruiz, Icaro P. Caruso, Bruna K. P. Sousa, Ana C. G. Jardim, Fabio C. L. Almeida, Laura H. V. G. Gil, Carolina H. Andrade, Glaucius Oliva

**Affiliations:** 1 Institute of Physics of Sao Carlos, University of Sao Paulo, Sao Carlos, SP Brazil; 2 Physical Sciences Laboratory, State University of Northern Rio de Janeiro Darcy Ribeiro (UENF), Campos dos Goytacazes, RJ Brazil; 3 Departament of Physics, Ribeirao Preto School of Philosophy, Science and Literature, University of Sao Paulo, Ribeirao Preto, SP Brazil; 4 Laboratory for Molecular Modeling and Drug Design, Labmol, Faculty of Pharmacy, Universidade Federal de Goiás, Goiânia, GO Brazil; 5 Institute of Biomedical Sciences, Federal University of Uberlandia, Uberlandia, MG Brazil; 6 Institute of Biosciences, Humanities and Exact Sciences (Ibilce), Sao Paulo State University (Unesp), Campus Sao Jose do Rio Preto, Sao Jose do Rio Preto, SP Brazil; 7 Institute of Medical Biochemistry (IBqM) Leopoldo de Meis, National Center of Nuclear Magnetic Resonance Jiri Jonas, Federal University of Rio de Janeiro, Rio de Janeiro, RJ Brazil; 8 National Center of Nuclear Magnetic Resonance (CNRMN), Center of Structural Biology and Bioimaging (CENABIO), Federal University of Rio de Janeiro, Rio de Janeiro, RJ Brazil; 9 Instituto Aggeu Magalhaes (IAM-FIOCRUZ), Recife, PE Brazil

**Keywords:** Biochemistry, Biophysics, Drug discovery

## Abstract

Chikungunya virus (CHIKV) is the causative agent of Chikungunya fever, an acute febrile and arthritogenic illness with no effective treatments available. The development of effective therapeutic strategies could be significantly accelerated with detailed knowledge of the molecular components behind CHIKV replication. However, drug discovery is hindered by our incomplete understanding of their main components. The RNA-dependent RNA-polymerase (nsP4-CHIKV) is considered the key enzyme of the CHIKV replication complex and a suitable target for antiviral therapy. Herein, the nsP4-CHIKV was extensively characterized through experimental and computational biophysical methods. In the search for new molecules against CHIKV, a compound designated LabMol-309 was identified as a strong ligand of the nsp4-CHIKV and mapped to bind to its active site. The antiviral activity of LabMol-309 was evaluated in cellular-based assays using a CHIKV replicon system and a reporter virus. In conclusion, this study highlights the biophysical features of nsP4-CHIKV and identifies a new compound as a promising antiviral agent against CHIKV infection.

## Introduction

The Chikungunya virus (CHIKV) belongs to the *Togaviridae* family and is the causative agent of Chikungunya fever. The main transmission route occurs through the bite of infected female mosquitoes of the *Aedes* sp. Genus. After CHIKV infection, the proportion of individuals who develop clinical and debilitating symptoms is considered the highest compared to other arboviruses, with an average of 80% of symptomatic cases^1,2^. The control of the mosquito vector remains the best prophylaxis since there are no licensed vaccines or efficient antivirals available^3^. In this scenario, the infection caused by CHIKV has a high social impact and constitutes a serious public health issue^3^.

Chikungunya fever presents an acute phase characterized by high fever, arthralgia, myalgia, headaches, edema, periorbital pain and cutaneous rash^4,5^. Later, some patients progress to the so-called chronic phase, mainly characterized by persistent arthralgia and musculoskeletal pain for months and even years^4,6–8^. Since there are no specific antiviral drug treatments, the clinical management targets primarily the relief of symptoms using analgesics and antipyretics in the acute phase; and non-steroidal anti-inflammatory drugs (NSAIDs) and corticosteroids in the chronic phase^5^.

CHIKV is a spherical, enveloped, and positive single-stranded RNA virus. As a member of the Alphavirus genus, its genome has approximately 12 kb and codes for two distinct polyproteins: non-structural and structural^9^. The first one is cleaved and gives rise to four non-structural proteins (nsP1, nsP2, nsP3 and nsP4) that form the viral replication complex and have functions in the infection process, such as interaction with host factors^9–11^. The nsP1 has methyltransferase and guanylyltransferase activities, promoting the capping of viral RNA. In addition, this protein also anchors the replication complex in the cell membrane^12^. The nsP2 is a multifunctional protein with NTPase and helicase activities in the N-terminal region. Its C-terminal has a cysteine-protease activity, responsible for processing the non-structural polyprotein^12^. The nsP3 is an accessory protein that recruits host cell factors that participate in and optimize the replicative process^13^. Finally, nsP4 is the RNA-dependent RNA polymerase (RdRp), which is considered the key enzyme of the CHIKV replication complex and acts principally by promoting the synthesis and elongation of viral RNA^14^.

On the other hand, the structural polyprotein is cleaved, giving rise to five structural proteins—E1, E2, E3, C and 6 k—which are part of the viral assembly and structure^9^. The envelope proteins, specially E2 and E1, are responsible for virus anchoring, receptor interaction and membrane fusion, promoting virus entry in the host cell^15,16^. Due to its location on the viral surface, envelope proteins are targets of the humoral immune response and thus become targets for vaccine development against CHIKV^17^.

Therefore, due to their vital role in the viral life cycle, non-structural proteins emerge as potential targets for developing antiviral drugs, aiming to interrupt the replication process and, consequently, the viral elimination^18^. Among these proteins, nsP4 is an attractive target due to its central role in viral genome replication, transcription and genome repair^14,19^. Recently, the three-dimensional structures of the RdRp domain of Sindbis (SINV) and Ross River (RRV) viruses has been experimentally solved^20^. However, the structure of this domain of CHIKV (nsP4-CHIKV) has not been reported. This fact generates limitations and challenges when the final goal is applying structure-based drug design strategies against this target.

Due to the lack of high-resolution structural information on the nsP4-CHIKV and the need to search for new drugs to treat the infection caused by the virus, we present here a detailed biophysical characterization of this protein. Size exclusion chromatography coupled with multi-angle light scattering was used to infer the oligomeric state of the nsp4-CHIKV protein in solution. A high prevalence of ordered helical secondary structures was observed by circular dichroism, which also showed that the nsP4-CHIKV unfolds under a cooperative transition during thermal denaturation. The thermal denaturation was further studied using differential scanning calorimetry, indicating a kinetically controlled process.

Moreover, in the search for ligands for the development of new inhibitors, we selected 12 compounds for an initial screen using differential scanning fluorimetry experiments. These compounds came from a global collaborative project named OpenZika^21,22^, through the World Community Grid computational network that enabled massive docking-based virtual screening campaigns for ZIKV proteins as well as other flaviviruses (DENV, YFV, WNV). One compound, LabMol-309, showed a significant effect on nsP4-CHIKV stability and its interaction with the protein was confirmed using a sophisticated combination of experimental and computational strategies.

Furthermore, the inhibitory activity of LabMol-309 was studied in cellular-based antiviral assays using a CHIKV replicon system and a reporter virus, and the results showed that this molecule caused inhibition in these cellular assays and has the potential to be further evaluated as a CHIKV inhibitor. In this way, this work provides novel structural features of nsP4-CHIKV and identifies a new compound that interacts with this protein, generating perspectives in the drug development field to treat the infection caused by CHIKV and potentially other alphaviruses.

## Results

### nsP4-CHIKV purification and SEC-MALS analysis

The nsP4-CHIKV was bacterially expressed and purified using chromatography systems, and the purity was confirmed by acrylamide gel electrophoresis (Supplementary Fig. 1). The nsP4-CHIKV is formed by 492 amino acids and has a theoretical molecular mass (MM) of 54.54 kDa. This construction covers the entire region of the RNA-dependent RNA polymerase (RdRp) domain, responsible for the nsP4-CHIKV function and where the catalytic aspartic acid residues (Asp371 and Asp466) are located^23^. The Asp466 is in the GDD motif, a highly conserved sequence of viral polymerases^23^.

**Figure 1:**
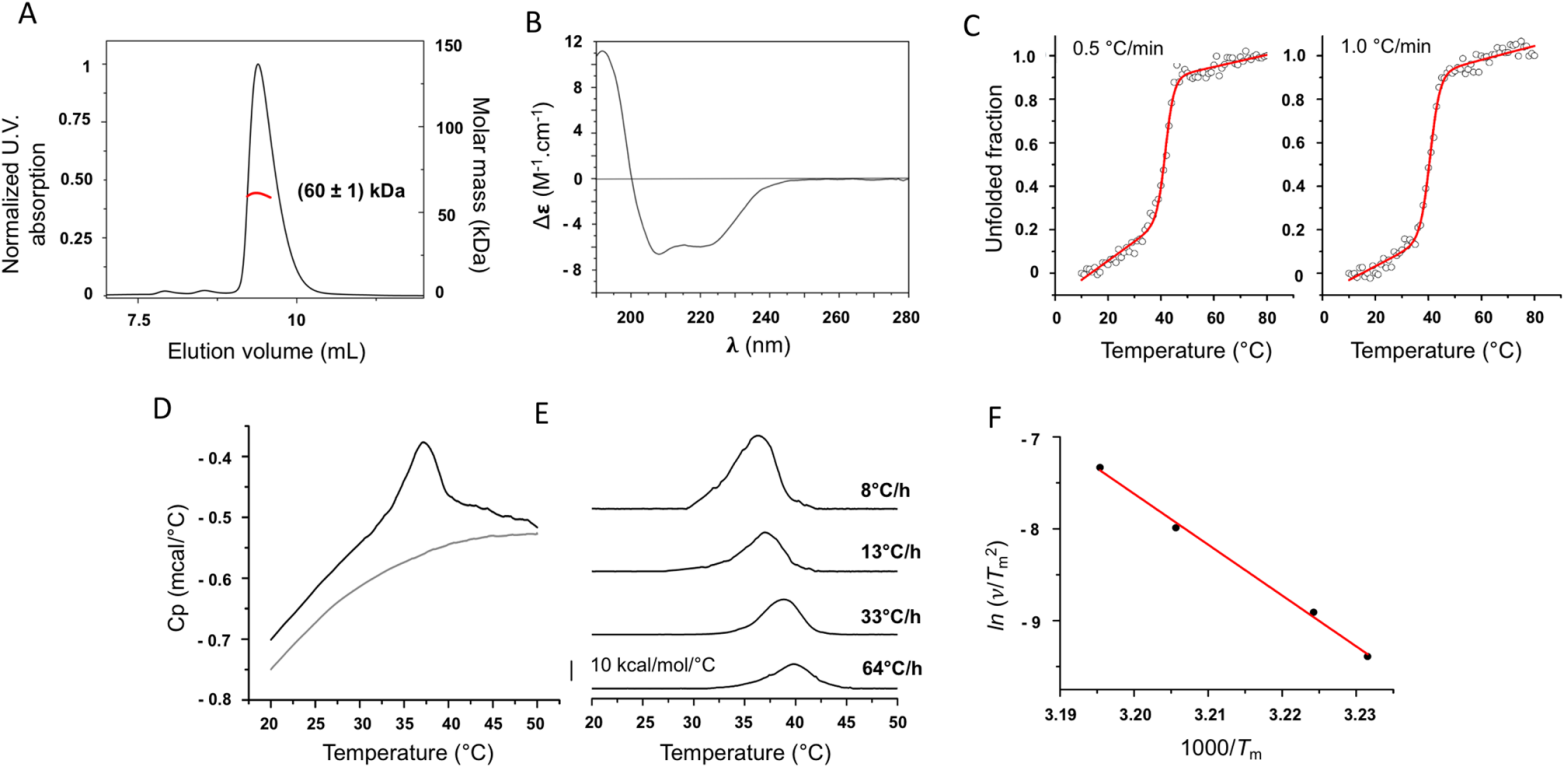
Biophysical characterization of nsP4-CHIKV. (**A**) SEC-MALS analysis of nsP4-CHIKV in solution. The proximity of the estimated molecular weight of (60 ± 1) kDa with the molecular weight of the protein suggests that nsP4-CHIKV is mostly at a monomeric state under the evaluated conditions. (**B**) Secondary structure profile of nsP4-CHIKV by CD spectroscopy. The nsP4-CHIKV spectrum suggests the predominance of helical secondary structures. (**C**) Thermal stability of nsP4-CHIKV probed by CD spectroscopy. The ellipticity at 222 nm as a function of temperature for nsP4-CHIKV was recorded at 0.5 °C/min (left) and 1.0 °C/min (right) and transformed to the protein unfolded fraction. Solid lines are best fits to the CD data using a two-state equilibrium model. The thermodynamic parameters of the protein unfolding transition are summarized in Table 1. (**D**) Scan-rate normalized (13 °C/h) DSC data of the irreversible thermal denaturation of 11.9 μM of nsP4-CHIKV and the corresponding instrumental buffer (50 mM Tris–HCl pH 8,0, 200 mM NaCl e 5% glycerol) baseline. (**E**) Excess heat capacity of nsP4-CHIKV at the indicated scan rates obtained after normalization by protein concentration and subtraction of the buffer baseline. (**F**) Arrhenius-type plot showing the scan rate dependence of the nsP4-CHIKV unfolding temperature, *T*_m_. The slope yields the activation energy for the irreversible denaturation of nsP4-CHIKV.

In order to determine the oligomeric state of nsP4-CHIKV in the working buffer solution, Size Exclusion Chromatography coupled with Multi-angle Light Scattering (SEC-MALS) was employed^24^. The SEC-MALS data showed a low polydispersity index and yielded a MM of $\left(60 \pm 1\right) kDa$ for nsP4-CHIKV (Fig. 1A). The proximity of the experimental value with the theoretical molecular mass of the protein suggests that the monomeric state is the most populated oligomer under the evaluated conditions.

### Evaluation of nsP4-CHIKV secondary structure profile

Circular dichroism (CD) spectroscopy was used to estimate the secondary structure content of nsP4-CHIKV in solution^25^. The CD spectrum of nsP4-CHIKV is characteristic of an α-helical rich protein, with two negative minima at 208 and 222 nm and one positive maximum around 195 nm (Fig. 1B). This profile corroborates with structural features described for viral polymerases and other polymerase structures solved experimentally^23^.

Dichroweb was used in the quantitative analysis of the CD spectrum^26^. The best-fit spectra were obtained with the CDSSRT method^27^, which displayed an NRMSD of 0.009 for the SP175 database and 0.012 for Sets 4 and 7, considered the most representative of the secondary structure content of nsP4-CHIKV (Supplementary Table 1). Secondary structure content estimation from the CD spectrum confirmed the higher percentage of α-helix in the nsP4-CHIKV (60%). The remaining fractions were: 6.3% of sheets, 9.6% of turns and 24% of disordered structure.

### nsP4-CHIKV thermal stability profile through CD spectroscopy and DSC

The CD technique was also applied to study the thermal behavior of this protein when subjected to temperature variations^28^. The unfolding transition of nsP4-CHIKV was studied by monitoring the ellipticity at 222 nm as a function of temperature^28^. The results showed that the thermal denaturation process occurred cooperatively, exhibiting the transition from folded to unfolded states in a defined way (Fig. 1C).

The thermodynamic parameters of the nsP4-CHIKV unfolding were obtained by adjusting a two-state equilibrium model to the experimental data. Changes in the heat capacity were not considered, and the fitting was performed taking into account the linear changes in pre- and post-transition ellipticity as a function of the temperature^28^. Thus, the melting temperature (*T*_*m*_), the apparent enthalpy change (*ΔH*_*app*_), and the apparent entropy change (*ΔS*_*app*_) for the same protein concentration were obtained at two different heating rates (Table 1).

**Table 1:** Thermodynamic parameters of nsP4-CHIKV unfolding by CD spectroscopy.

Rate (°C/min)	*T*_*m*_ (°C)	Δ*H*_*app*_ (kcal/mol)	Δ*S*_*app*_ (cal/mol K)
0.5	41.8 ± 0.2	122 ± 12	387 ± 38
1.0	43.7 ± 0.2	104 ± 8	331 ± 26

The *T*_m_ and the Δ*H*_app_ were determined by fitting the CD data to a two-state equilibrium model^28^. The Δ*S*_app_ of the unfolding transition at *T*_m_ was calculated as Δ*S*_app_ = Δ*H*_app_/*T*_m_ since Δ*G* = 0 at *T*_m_.

The thermodynamic parameters of the nsP4-CHIKV unfolding transition exhibited a dependence on the heating rate. This result suggests that the irreversible nsP4-CHIKV unfolding transition is kinetically dependent^29,30^. The values of the enthalpy and entropy changes obtained from the CD data agree well with those observed for globular proteins^31^. The thermal behavior of nsP4-CHIKV was also evaluated using Differential Scanning Calorimetry (DSC)^32^. Figure 1D shows the temperature-dependence of the heat capacity profile (*C*_p_ – sample minus reference) of nsP4-CHIKV and the instrumental buffer baseline, acquired with a scan rate of 13 °C/h. The protein *C*_p_ was subtracted from the buffer baseline *C*_p_ and normalized to the protein concentration.

DSC experiments were performed at different scan rates to investigate its effect on the DSC profile and the reversibility of the transitions. The temperature- and scan rate dependence of the excess heat capacity profile of nsP4-CHIKV are illustrated in Fig. 1E. The nsP4-CHIKV undergoes an irreversible thermal denaturation, and the values of the calorimetric enthalpy change (Δ*H*_*cal*_*)* obtained from the analysis of the thermograms are within the range of values observed for other globular proteins^33–36^. Moreover, the transition peak shows a clear scan rate dependence, confirming that all thermodynamic parameters associated with nsP4-CHIKV thermal denaturation depend upon the heating rate (Table 2), as observed in our previous CD analysis. Except for the van't Hoff enthalpy change (Δ*H*_vH_), the dependences of the thermodynamic parameters on the heating rate are markedly non-linear (Table 2 and Supplementary Fig. 2). This feature illustrates the non-equilibrium character of the protein denaturation process.

**Table 2:** Thermodynamic parameters associated with the nsP4-CHIKV thermal denaturation by DSC.

Concentration (µM)	Rate (°C/h)	*T*_*m*_ (°C)	Δ*H*_*cal*_ (kcal/mol)	Δ*T*_*1/2*_ (°C)	Δ*S* (cal/mol)	Δ*H*_*vH*_ (kcal/mol)
9.3	8	36.3	205	4.8	662	142
11.9	13	37.0	102	4.4	330	151
7.4	33	38.8	86	4.3	275	165
7.4	64	39.8	66	4.5	210	150

*T*_*m*_ represents the temperature where *C*_P_ reaches its maximum value. Δ*H*_*cal*_ was calculated as the area under the DSC trace. Δ*T*_*1/2*_ corresponds to the linewidth at half the height of the transition peak. The entropy change at *T*_m_, Δ*S*, was calculated as Δ*S* = Δ*H*_cal_/*T*_m_. Δ*H*_vH_ was calculated as 4*RT*_m_^2^*C*_p,max_/Δ*H*_cal_. Analyses of the thermograms were performed with MicroCal Origin software.

Uncertainties: *T*_*m*_ (± 0.2 °C), Δ*H*_*cal*_ (± 1‒3 kcal/mol), Δ*T*_*1/2*_ (± 0.2 °C).

The dependence of *T*_m_ on the scan rate was used to calculate the kinetic activation energy, *E*_a_, for the irreversible nsP4-CHIKV thermal denaturation. According to Sanchez-Ruiz et al.^30^, the *T*_m_ shifts induced by different heating scan rates, ν, for an irreversible two-state process can be modeled by the following equation:

$$\frac{\nu }{{T_{m}^{2} }} = \frac{AR}{{E_{a} }}e^{{ - \frac{{E_{a} }}{{RT_{m} }}}}$$

where A is the pre-exponential factor in the Arrhenius equation, and *R* is the gas constant. Thus, by plotting $ln\left(\nu /{T}_{m}^{2}\right)$ against 1/*T*_m_, the apparent activation energy can be determined from the slope of the curve. The Arrhenius plot showing the scan rate-dependent changes in the *T*_m_ is illustrated in Fig. 1F, from which *E*_a_ was determined as (110 ± 4) kcal/mol.

### Evaluation of nsp4-CHIKV interaction with compounds

**Figure 2:**
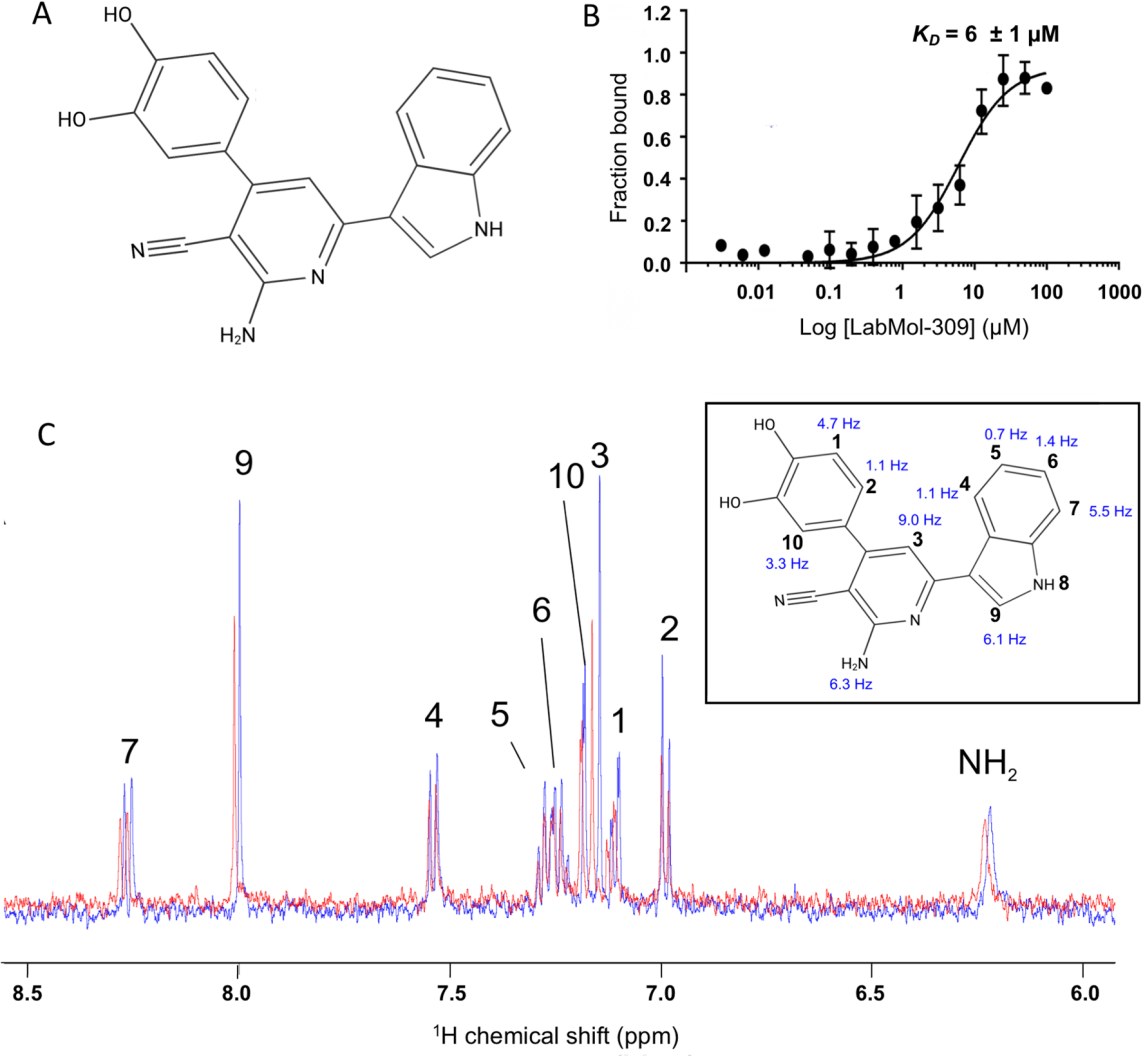
Evaluation of nsp4-CHIKV interaction with the compound LabMol-309. (**A**) LabMol-309 chemical structure. (**B**) Binding affinity curve of nsP4-CHIKV interacting with LabMol-309 by MST. The compound LabMol-309 was titrated in a concentration range of 200 µM to 0.012 µM. The curve was fitted to the Hill function, and the estimated *K*_*D*_ was (6 ± 1 µM). (**C**) Chemical shift perturbation analysis based on the superimposition of LabMol-309 one-dimensional 1H spectra obtained in the presence (red line) and the absence of nsP4-CHIKV (blue line). On the right, chemical shift differences were measured (in Hz) for each proton of the LabMol-309 structure.

In the search for new compounds able to interact and inhibit the nsP4-CHIKV in solution, an initial experimental screening with a series of 12 compounds (Supplementary Table 2) was selected from the OpenZika project^22,37^ and performed using differential scanning fluorimetry (DSF or ThermoFluor assay). For nsP4-CHIKV, the *T*_*m*_ in the absence of compounds (only with DMSO) was 37.7 ± 0.4 °C. Among the compounds, LabMol-309 (Fig. 2A) caused the highest thermal shift (~ 4 °C), suggesting the occurrence of interaction with nsP4-CHIKV. Therefore, this compound was chosen to proceed with the other assays.

The interaction between LabMol-309 and nsP4-CHIKV was later analyzed by MicroScale thermophoresis (MST) and solution nuclear magnetic resonance (NMR). The MST data showed an affinity curve with the occurrence of well-defined bound and unbound states (Fig. 2B). From that, the dissociation constant (*K*_*D*_) for the interaction of nsP4-CHIKV with LabMol-309 was estimated as (6 ± 1) µM.

The interaction of LabMol-309 with nsP4-CHIKV was further evaluated by solution NMR, monitoring the chemical shift perturbation (CSP). Figure 2C shows the spectra obtained for the compound LabMol-309 in the presence (red line) and absence of the protein (blue line). These spectra were superimposed, and the chemical shift differences were identified and mapped according to the respective positions of the proton resonances (Fig. 2C) previously identified in the LabMol-309 assignment (Supplementary Fig. 3).

Therefore, detecting these chemical shifts perturbations is additional evidence for the interaction between nsP4-CHIKV and compound LabMol-309, reinforcing the results obtained using DSF and MST.

### The nsP4-CHIKV three-dimensional model and structural analysis

**Figure 3:**
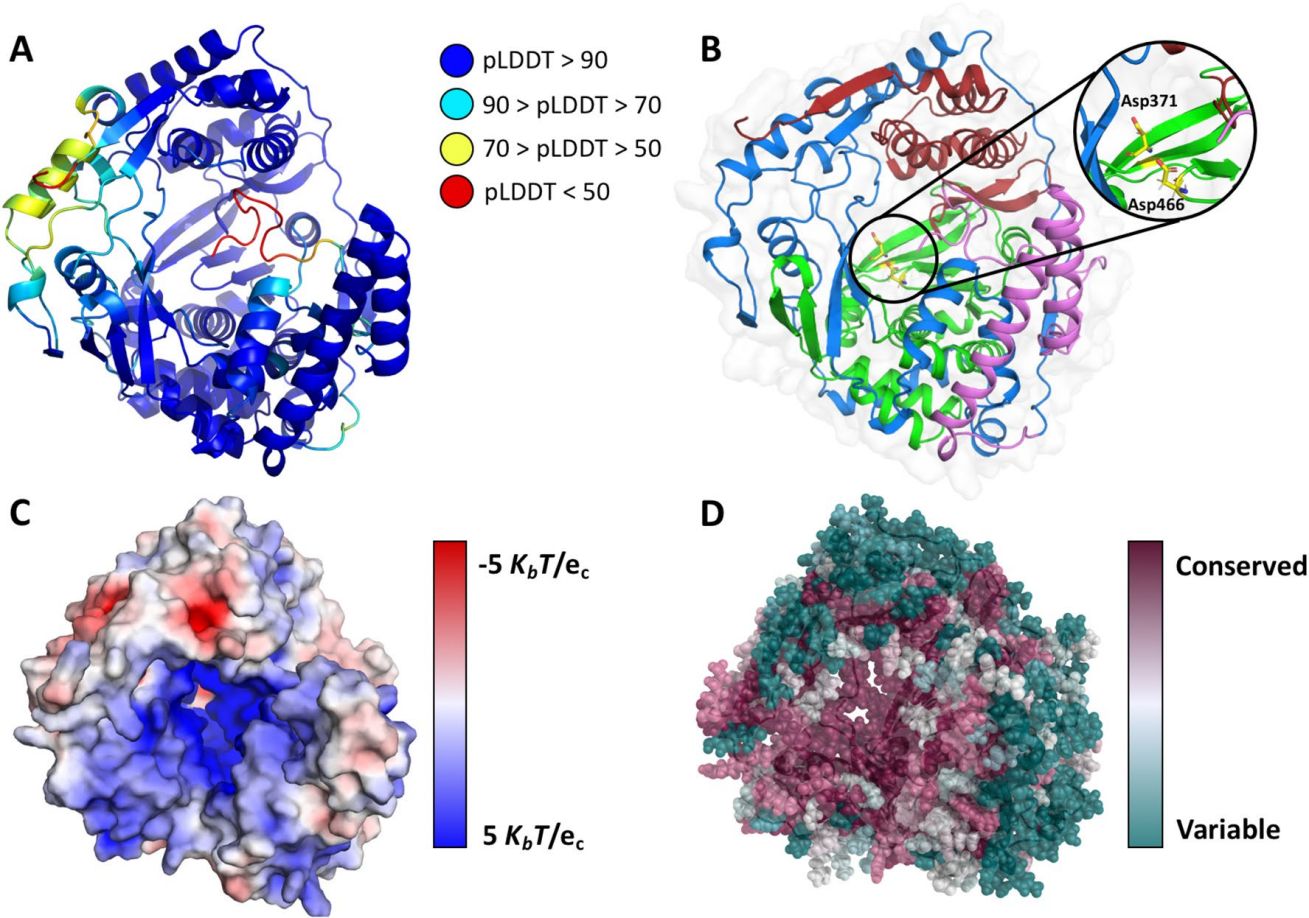
nsP4-CHIKV three-dimensional model by AlphaFold. (**A**) nsP4-CHIKV AlphaFold model colored according to pLDDT. At the top right are shown the pLDDT color scales. (**B**) Model of nsP4-CHIKV colored according to domain assignment. The N-terminal helixes, *finger, palm* and *thumb* domains are colored in purple, dark blue, green and red, respectively. A detailed view of predicted catalytic aspartic residues is presented in the inset. (**C**) Electrostatic potential projected on the surface charge of nsP4-CHIKV, calculated with APBS. Positive regions are colored in blue and negative regions are in red. (**D**) ConSurf analysis of nsP4-CHIKV model. The amino acids are colored by their conservation grades using the color-coding bar, with green-through-purple indicating variable-through-conserved.

A combined analysis of several biophysical techniques suggests that the nsP4-CHIKV is a monomeric α-helical rich protein capable of forming a complex with the compound LabMol-309 within a moderate dissociation constant (10^−6^ M). The nsP4-CHIKV 3D structural model was obtained using AlphaFold^38^. The model obtained showed a very high per-residue confidence score (pLDDT) for more than 90% of the covered sequence, and only for the N- and C-terminal regions pLDDT were low (Fig. 3A). The full-length nsP4-CHIKV structural model is illustrated in Fig. 3.

In the nsP4-CHIKV structure, we identified the regions corresponding to the *fingers* (residues 93–315), *palm* (residues 316–502) and *thumb* (residues 503–611) domains, which are characteristics of viral polymerases (Fig. 3B). In the palm domain's catalytic site, we also identified the catalytic aspartic acid dyad (Asp371 and Asp466) separated by 7.5 Å. Besides, the nsP4-CHIKV model contains an extra N-terminal domain (*residues 1–92),* forming a coiled-coil.

In the model of nsP4-CHIKV, the electrostatic surface potential of the active site cavity where are located the aspartic acid dyad (Asp371 and Asp466) is remarkably positive, a signature of nucleic acids-interacting motifs (Fig. 3C)^39^. Additionally, the analysis using ConSurf reveals that the catalytic site region and its surroundings are highly conserved (Fig. 3D). These results serve as corroboration for the robustness of the AlphaFold model of nsP4-CHIKV.

### Molecular docking and molecular dynamics of LabMol-309 against nsP4-CHIKV

**Figure 4:**
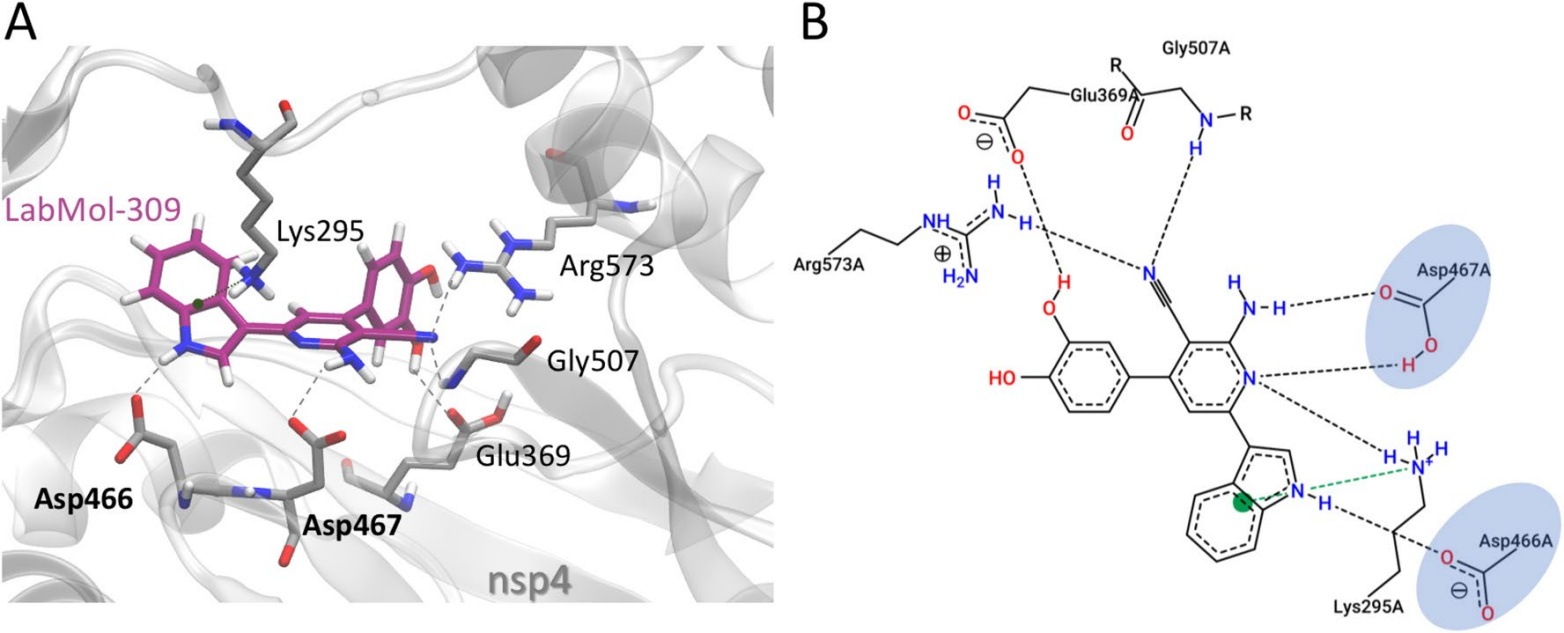
Molecular interactions of LabMol-309 and nsP4-CHIKV, predicted by docking calculations. (**A**) 3D interactions of LabMol-309 and nsP4-CHIKV residues. Hydrogen bonds are shown as gray dashed lines, and cation–π interactions are shown in green dashed lines. Oxygen, nitrogen and hydrogen atoms are shown in red, blue and white, respectively. Carbon atoms of LabMol-309 and protein residues are shown in purple and gray, respectively. Catalytic residues of the GDD triad are highlighted in bold. (**B**) 2D interaction diagram of LabMol-309 and nsP4-CHIKV residues. Hydrogen bonds are shown as gray dashed lines, and cation–π interactions are shown in green dashed lines. Catalytic residues of the GDD triad are highlighted in blue.

Docking calculations were used to investigate the binding mode of LabMol-309 at the nsP4-CHIKV. The docking results suggest that LabMol-309 binds to the nsP4-CHIKV active site, interacting with the GDD catalytic triad (Asp466 and Asp467), with a docking score of -7.15 kcal/mol^−1^. LabMol-309 makes H-bonds with Glu369, Asp466, Asp467, Gly507, Arg573 and cation–π interactions with Lys295 residue (Fig. 4). The nitrogen atom of the indole group and amine of the pyridine group of LabMol-309 make relevant interactions with Asp466 and Asp467, respectively. Additionally, the indole group makes cation–π interaction with Lys295.

**Figure 5:**
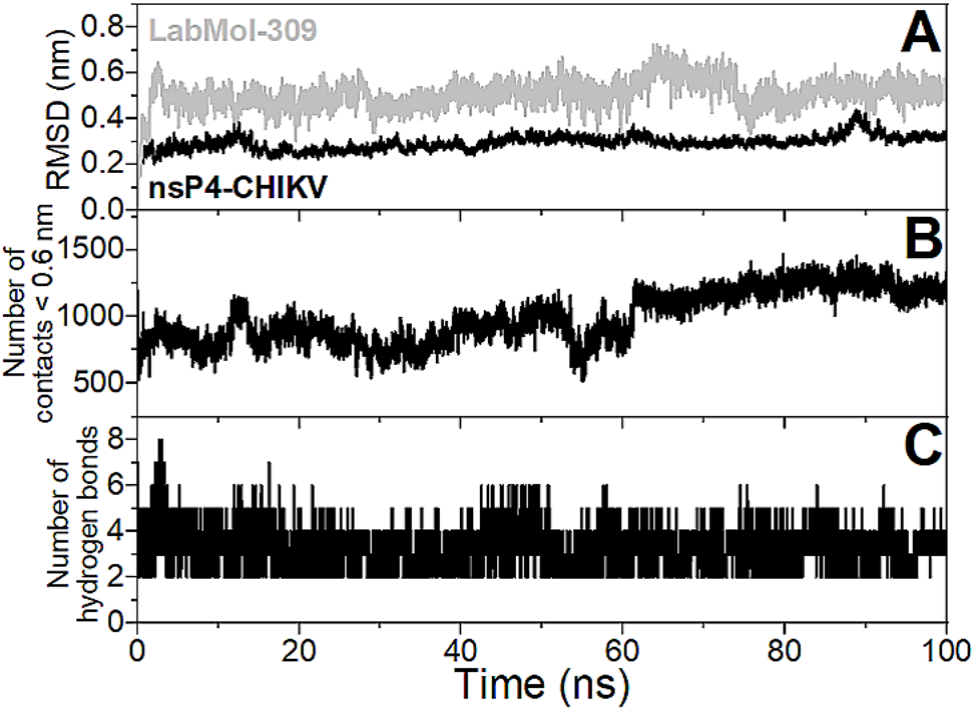
Evaluation of the stability of the structural model of the nsP4-CHIKV/LabMol-309 complex from 100 ns MD simulation. (**A**) RMSDs of backbone atoms of the protein (black line) and non-hydrogen atoms of the ligand (gray line). (**B**) Number of contacts between atoms of the protein and ligand for distances less than 0.6 nm. (**C**) Number of hydrogen bonds formed between the protein and ligand.

The structural stability of the structural model of the nsP4-CHIKV/LabMol-309 complex calculated by docking was evaluated using 100 ns molecular dynamics (MD) simulation. Figure 5A presents the values of root mean square deviation (RMSD) for the backbone atoms of the protein and non-hydrogen atoms of the ligand from the initial structure. It is possible to observe that RMSD values are stable after 5 ns of simulation and reach plateaus around 0.3 and 0.5 nm for the protein and ligand, respectively. Figure 5B shows that the number of contacts < 0.6 nm between nsP4-CHIKV and LabMol-309 does not drop down to zero throughout the MD simulation, indicating that the ligand interacts with the protein is persistent. The number of hydrogen bonds is stable throughout the MD simulations and presents an average value of three (Fig. 5C). An evaluation of the hydrogen bonds with significant percentage occupancy (< 5%, Supplementary Table 3) during the MD trajectory reveals that Glu369, Asp371, Asp466, Asp467, Asn468, Lys501, and Arg573 are important for the stabilization of the protein–ligand complex, and further mutagenesis studies may be relevant for confirmation of these interactions. It is worth noting that Glu369 and Asp371 presented percentage occupancies higher than 70%. Considering all MD analyses, it can be concluded that the structural model of the nsP4-CHIKV/LabMol-309 complex is stable throughout the simulation.

### Inhibition of CHIKV replication by LabMol-309 through replication-based and viral infection assays

**Figure 6:**
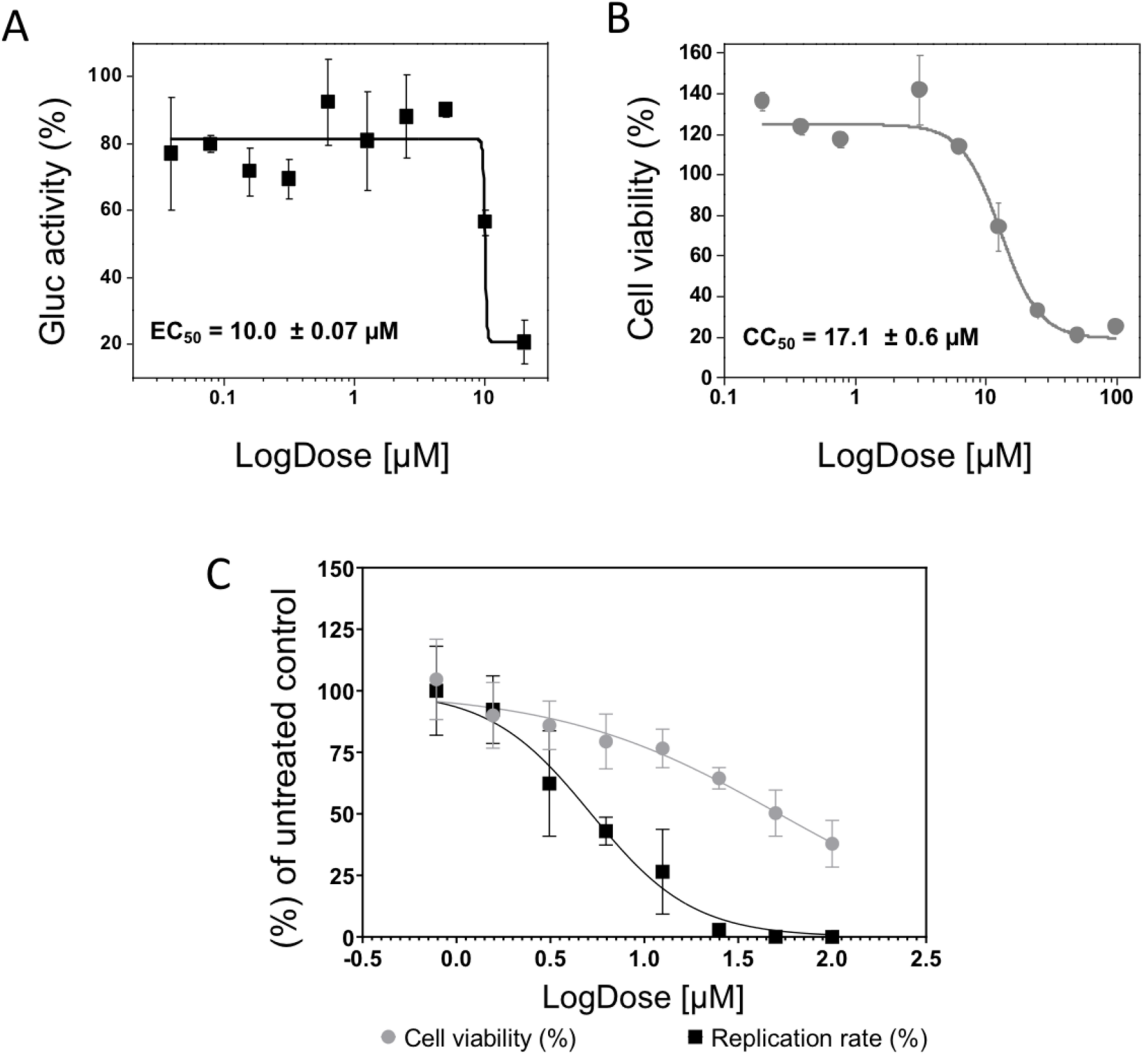
Antiviral activity of LabMol-309. The EC_50_ (**A**) and CC_50_ (**B**) curves from replicon-based assays are shown. CHIKV replicon cells were incubated with the compound at twofold serial dilutions (from 20 µM to 0.03 µM for EC_50_ and from 100 to 0.3 µM for CC_50_) for 48 h, and Gluc activity/cell viability were measured from cells' supernatant. For the CHIKV-*nanoluc* replication assay evaluation of EC_50_ and CC_50_ (**C**), BHK-21 cells were treated with concentrations of LabMol-309 ranging from 0.78 to 100 µM, in the presence or absence of CHIKV-*nanoluc* for 16 h, and viral replication was quantified by measuring the nanoluciferase activity (indicated by ●) and cellular viability was measured using an MTT assay (indicated by ^▄^). Representative results from two independent experiments performed in duplicates. Error bars represent the standard deviations. Figures and statistical analysis were performed using GraphPad Prism 8.

The inhibitory activity of the compound LabMol-309 was evaluated through the replicon-based screenings in a dose-dependent manner to determine its effective and cytotoxic concentrations (EC_50_ and CC_50_, respectively). Replicon cells were incubated with twofold serial dilutions of compound (from 20 to 0.03 µM for EC_50_ and from 100 to 0.30 µM for CC_50_), and luciferase signals or cell viability was evaluated after 48 h. LabMol-309 displayed an EC_50_ value of 10.0 ± 0.07 µM and showed a CC_50_ of 17.1 ± 0.6 µM (Fig. 6; Supplementary Table 4 and 5). As a result, the obtained selectivity index (CC_50_/EC_50_) of LabMol-309 was 1.7 in the replicon system.

To confirm the antiviral activity of the LabMol-309, we carry out the effective concentration of 50% (EC_50_) and cytotoxic concentration of 50% (CC_50_) using BHK-21 cells infected with CHIKV-*nanoluciferase*, a recombinant CHIKV that express the Nanoluciferase reporter, at a multiplicity of infection (MOI) of 0.1, with a two-fold serial dilution of LabMol-309 at concentrations ranging from 0.78 to 100 µM. N*anoluciferase* activity levels, proportional to viral replication, were assessed 16 h post-infection (adapted from^40,41^). The cytotoxic concentration of 50% (CC_50_) was determined in parallel experiments (Fig. 6C). As a result, these assays demonstrated that the LabMol-309 has a EC_50_ of 5.2 µM on BHK-21 cells infected with CHIKV-*nanoluc* and CC_50_ of 52 µM on naive BHK-21 cells, over a period of incubation of 48 h, resulting in a Selectivity Index (SI) of 10 (Table 3; Fig. 6C; Supplementary Table 6).

**Table 3:** Effect of the compound on the viability of BHK-21 cells (CC_50_) and the viral replication of CHIKV (EC_50_) in 16 h treatment.

Compound	Type of the Assay	CC_50_	EC_50_	Selectivity index (SI)
LabMol-309	CHIKV-*nanoluc* and BHK-21 cells	52	5.2	10
	BHK-CHIKV replicon	17.1	10.0	1.7

## Discussion

The nsP4-CHIKV polymerase plays a crucial role in viral replication and has been considered a promising target for the search and development of new drugs. Thus, understanding its dynamic and structural features is an important step for studies with this target.

Our biophysical data agree with the structure prediction using Alphafold and point out that nsp4-CHIKV is a monomeric protein enriched with alpha-helix content. These observations also agree with experimental data collected for other viral polymerases^20,23^. The thermodynamic data showed that when in the in vivo host, this protein can be stable in the vicinity of its thermal denaturation (T_m_ ~ 40 °C and ΔT_*1/2*_ ~ 4–5 °C), and its unfolding is both scan-rate dependent and irreversible under all conditions tested. It was shown before that the T_m_ might be scan-rate dependent if the scan rate exceeds the unfolding rate^42^. The calorimetric thermograms for the irreversible denaturation of proteins are highly scan-rate dependent, and their shapes are normally asymmetric^43^, exactly what is observed for nsp4-CHIKV. Therefore, the kinetics of the thermal denaturation could be treated as a single first-order irreversible step N → D, whose rate of temperature dependence obeys the Arrhenius equation. The effective activation energy derived from this equation was (110 ± 4) kcal/mol, which fits well within the wide range of the values reported for the thermal denaturation of mammalian tissues and strengthened the thermodynamic data collected for this protein^44^.

In the search for new nsp4-CHIKV ligands as potential inhibitors, the compound LabMol-309 was identified as a promising candidate through DSF screening. The validation of this interaction through biophysical methods demonstrated that the compound interacts in the low micromolar range. This compound had already been evaluated in virtual screenings for other viral proteins such as ZIKV and other Flaviviruses^21,22^, but it was the first time it was reported targeting the nsP4-CHIKV protein.

A three-dimensional model was generated using AlphaFold to analyze the LabMol-309-nsP4-CHIKV complex and their mode of interaction. In this sense, the nsP4-CHIKV model presented the regions corresponding to the *fingers*, *palm* and *thumb* domains, which are characteristics of viral polymerases, and the active site region was remarkably positive and conserved. These structural features were equivalent to the regions presented in the RdRp domain of nsP4 from both RRV and SINV, which were experimentally solved recently (PDB ID: 7F0S, 7VB4, 7VW5)^20^. Therefore, the suggested binding mode for LabMol-309 is through the interaction with residues of the nsP4-CHIKV active site.

Although the data involving the complex formation between nsP4-CHIKV and LabMol-309 are solid, it is still not possible to conclude that this compound has inhibitory activity against this enzyme. The development of an enzymatic assay for the nsP4 polymerase from Alphaviruses in general, has been challenging because this protein cannot effectively perform its function on its own, as previously shown by others^45,46^. Different regions of nsP4 recognize the promoters for *minus* and *plus* strands. However, the binding requires the presence of the other non-structural proteins to form the replication complex and enable the de novo RNA synthesis^11,45–47^. Moreover, the interactions between these proteins with host components during replication have been studied but remain limited and not completely understood^11,46^.

Tomar et al.^48^ reported that template recognition and the nsP4 activation through protein–protein interactions requires the presence of viral polyprotein P123^48^. In another work, the SINV nsP4 was expressed in *E. coli,* and the polymerase activity was observed only when supplied with the viral polyprotein P123^47^. Recently, Lello et al.^46^ demonstrated that nsP4 of SINV, CHIKV, ONNV, BFV, RRV, SFV, MAYV, VEEV, and EILV on their own have minimal RNA polymerase activity^46^. Using a trans replicase system consisting of two relatively independent functional modules (nsP4 and P123), they have shown that the nsP4 of all these Alphaviruses was active only when combined with the corresponding P123 polyproteins^46^. Furthermore, Tan et al.^20^ evaluated the polymerase activity of SINV and RRV nsP4 and as a result, the isolated proteins showed less efficient polymerase activity than the dengue virus RdRp used as the positive control^20^. Altogether these findings corroborate that bacterially produced nsP4 could not efficiently synthesise RNA unless combined with the viral polyprotein P123 obtained from animal cell extracts^47,49,50^.

Given these limitations in establishing an efficient method for evaluating the enzymatic activity of purified recombinant nsP4-CHIKV, in this work the inhibitory effect of the compound LabMol-309 was evaluated using both replicon-based assays and cells infected with the CHIKV expressing the nanoluciferase reporter (CHIKV-nanoluc). Replicon-based systems have been widely used as tools for drug discovery of antiviral agents, and promising replication inhibitors were identified by this method^51^. Specifically to CHIKV, BHK-21 cells harboring other CHIKV replicon constructs were reported for the high-throughput screening of viral replication inhibitors^52,53^. The same system was also used to evaluate the anti-CHIKV activity of other compounds and different flavonoids^54–56^.

The evaluation of LabMol-309 using a replicon-based system was performed in a dose-dependent manner, and its inhibition clearly occurred. Comparing with studies that also used CHIKV replicon, the EC_50_ obtained for LabMol-309 was lower than the values already reported for other compounds^52,55^, reinforcing the antiviral potential of this compound. LabMol-309 showed toxicity to the cells, and the resulting low selectivity index of 1.7 may be correlated to a possible negative impact in the cellular factors associated with the viral genome replication. These data suggest that, even with inhibitory activity, chemical modifications would be required to optimize this compound's efficiency and reduce its toxicity. Furthermore, antiviral assays performed with cells infected with a recombinant CHIKV demonstrated that the LabMol-309 decreased CHIKV replication with an EC_50_ of 5.2 µM and an CC_50_ of 52 µM, with a SI of 10 in BHK-21 cells.

The differences in the obtained values using naive BHK-21 or BHK-CHIKV cells (Table 3) are understandable since different factors are involved in these assays. For example, in the infection system the virus is effectively infecting the cells and performing all the stages of the virus replicative cycle. It means that the treatment with LabMol-309 may be acting even before the formation of the replication complexes. Alternatively, in the BHK-CHIKV replicon system, the replication complexes are already formed when the treatment starts, which can impact on the effectiveness of the antiviral activity in a short period of treatment. Additionally, the presence of the replicon might change the cell response to the compound, and explain the higher cytotoxicity shown in the results. This isolated effect predominantly observed in the replicon cells can be explained by the differences in incubation periods used in the antiviral activity experiments (48 h for replicon-based screenings compared to 16 h for the viral infection assays). The prolonged exposure of cells to the compound can result in higher cytotoxicity^57^, reinforcing the importance of further studies of the ADME-Tox profile in animal models. Additionally, to the best of our knowledge, this is the first description of LabMol-309 as inhibitor of CHIKV replication, and its low EC_50_ value is in similar level with other inhibitors reported to block CHIKV replication, emphasizing the antiviral potential of this compound^58,59^. In this context, the results obtained from the antiviral assays suggest that LabMol-309 is a potential molecule to be further optimized to reduce its cytotoxicity and increase the selectivity index in cell-based antiviral assays. In summary, this study highlights biophysical features of nsP4-CHIKV, contributing to basic research on alphaviruses polymerase, and identified a new compound as a promising antiviral agent against CHIKV infection. These findings could contribute to developing novel candidates targeting nsP4-CHIKV and support the progress in therapeutic strategies for CHIKV and other alphavirus infections.

## Methods

### CHIKV nsP4 cloning and overexpression

The coding region of nsP4-CHIKV (GenBank KP164572.1; PROT-ID AJY53709.1—residues 118 to 611) was subcloned in the pET-SUMO vector using the LIC methodology^60^. This construct encodes an nsP4-CHIKV with an N-terminal 6xHis-tag followed by a TEV protease cleavage site (ENLYFQ; GAM) and the fusion protein tag SUMO. For protein expression, this plasmid construction was transformed into *E. coli* Rosetta and cultured in Terrific Broth (TB) medium at 37 °C and 200 RPM until an OD_600_ of 1.0. The protein expression was induced with 1 mM of isopropyl-β-thiogalactoside and incubation at 18 °C, 200 RPM for 16 h. The cells were harvested by centrifugation at 5000 × *g* for 30 min at 4 °C and resuspended in buffer A (50 mM Tris pH 8.0, 500 mM NaCl, 10% glycerol). Cells were disrupted by sonication and clarified by centrifugation at 12,000 × *g* for 30 min at 4 °C.

### nsP4-CHIKV purification

nsP4-CHIKV was purified using an AKTA Purifier System (GE Healthcare). The first step was affinity chromatography, using a HisTrap HP 5.0 mL column (GE Healthcare) pre-equilibrated with buffer A (50 mM Tris pH 8.0, 500 mM NaCl, 10% glycerol). The elution was performed using 50 mM Tris pH 8.0, 500 mM NaCl, 250 mM imidazole, 10% glycerol. The buffer was exchanged through dialysis to eliminate the imidazole excess. The 6xHis-tag-SUMO was cleaved by TEV protease during overnight incubation at 4 °C. A second affinity chromatography step was performed using the same system to collect the HisTag-less protein obtained after TEV treatment. A final purification step was done using size-exclusion chromatography on an XK 26/1000 Superdex 75 column (GE Healthcare) pre-equilibrated in gel filtration buffer (50 mM Tris pH 8.0, 200 mM NaCl and 5% glycerol). The eluted fractions were collected and analyzed by SDS-PAGE to confirm their purity and mass spectrometry was performed to confirm the presence of nsP4-CHIKV. The final protein sample was concentrated using 30 kDa MWCO centrifugal concentrators (Vivaspin, Sartorius). Protein concentrations were determined spectrophotometrically in a Nanodrop 1000 spectrophotometer, using the measured absorbances at 280 nm and the theoretical extinction coefficient of 36,495 M^−1^ cm^−1^.

### Size exclusion chromatography coupled with multi-angle light scattering (SEC-MALS)

The oligomeric state of the purified nsP4-CHIKV was evaluated by size exclusion chromatography coupled with multi-angle light scattering (SEC-MALS) in running buffer composed of 50 mM Tris–HCl pH 8.0 and 200 mM NaCl. For that, 50 µL of purified nsP4-CHIKV at a concentration of 1.5 mg/mL was injected in a Waters 600 HPLC system (Waters) coupled in-line with a UV detector, a mini DAWN TREOS multi-angle light scattering apparatus (Wyatt Technology), a column Superdex 75 Increase 10/300 GL (GE Healthcare), and a refractive index detector Optilab T-rEX (Wyatt Technology). The light scattering detectors were normalized with bovine serum albumin (Sigma-Aldrich) and the flow rate used was 0.5 mL/min. The data were processed using ASTRA7 software (Wyatt Technology) with the following parameters: refractive index of 1.331, 0.890 cP for the viscosity of the solvent, and a refractive index increment of 0.1850 mL/g. Protein solutions were centrifuged for 10 min at 10,000 × *g* at a controlled temperature of 4 °C immediately before use.

### Circular dichroism (CD)

Far UV-CD spectra (195–280 nm) were measured in a Jasco J-810 spectrometer (Jasco Corporation, Japan) equipped with a Peltier control system and using a quartz cell with a 1 mm pathlength. The spectra were recorded from 280 to 195 nm, with a scanning speed of 100 nm/min, a spectral bandwidth of 1 nm and a response time of 0.5 s. All the protein samples were in a final concentration of 2.5 µM diluted in water. Spectral deconvolution was applied to estimate the secondary structure content using the DICHROWEB web server^26^. Three different methods were combined with three different databases to improve the reliability of the results. The detailed analysis of the results generated by these combinations is provided in Supplementary material (Supplementary Table 1). The estimated values of secondary structure fractions were averaged from each database used. The best fit was determined from the analysis of the NRMSD parameter, which was considered satisfactory when closer to 0^61^. Thermal denaturation experiments were performed by monitoring the ellipticity at 222 nm in the range from 20 to 80 °C using heating rates of 0.5 °C/min and 1.0 °C/min.

### Differential scanning calorimetry (DSC)

DSC measurements were carried out with the purified protein solution at 7.4 µM, 9.3 µM and 12 µM, diluted in buffer 50 mM Tris–HCl (pH 8.0), 200 mM NaCl and 5% glycerol. Protein and reference samples (buffer) were degassed for 5 min prior to measurements. The experiments were performed on a VP-DSC MicroCal MicroCalorimeter (Microcal, Northampton, MA, USA) using heating rates of 8 °C/h, 13 °C/h, 33 °C/h and 64 °C/h. The thermograms were recorded from 10 to 70 °C, at a controlled pressure of 1.6 atm. Instrumental buffer baselines were recorded before the protein unfolding experiments to register the thermal history of the calorimeter. The raw DSC traces were subtracted with the buffer baseline and then normalized by protein concentration. The thermogram analysis and the subtraction of the buffer calorimetric response, baseline correction, and integration of the calorimetric peaks referring to the phase transitions were performed using the MicroCal Origin software.

### Differential scanning fluorimetry (DSF)

In the search for new binders to nsp4-CHIKV, 12 compounds from the OpenZika project were tested^22,37^. The compounds were purchased from Chembridge Library (https://www.chembridge.com/) with a minimum purity of 90%. DSF assays were conducted in a qPCR system Mx3000P (Agilent) for an initial assessment. Protein melting temperatures (*T*_*m*_), assuming a two-state transition model, were determined by monitoring the fluorescence intensity variation as a function of temperature for the extrinsic probe SYPRO Orange (Invitrogen). The protein solutions were at a final concentration of 8 µM, diluted in gel filtration buffer. Compounds were added at the final concentration of 80 µM and standard samples were prepared only with DMSO. The thermal variations were in the range of 25–75 °C in a stepwise increment of 1 °C/min. The *T*_*m*_ values obtained for each compound were subtracted from the values of the standard samples to identify compounds that caused significant *T*_*m*_ changes. For the next steps, the compound that presented the highest thermal shifts (Δ*T*_*m*_) was selected, considering the deviations of the triplicates^62^. Data were analysed using the software Origin Pro 9.5.1. All experiments were conducted in triplicate.

### MicroScale thermophoresis (MST)

Experiments were performed on a Monolith® NT.115 instrument (Nanotemper technologies). The nsP4-CHIKV was labelled on cysteine residues with NT-647-Maleimide dye (Nanotemper Technologies) using the Monolith NTTM Protein Labeling Kit RED-MALEIMIDE as per manufacturer's instructions. Samples of 25 nM of cys-labelled nsP4-CHIKV with 5% DMSO were used. An initial binding test was carried out with the compound at the concentration of 100 µM, to check the interaction between the protein and the compound. Then, a serial dilution of the compound from 200 to 0.012 µM (12 nM) was performed to obtain the binding curve. The dissociation constant (*K*_*D*_) was obtained by fitting the binding curve with the Hill function using GraphPad Prism 8 (Graph Pad Software).

### Chemical shift perturbation

The LabMol-309 resonance assignment was performed using a Bruker Avance III 600 MHz. 1H-13C-HSQC, COSY and TOCSY were acquired at 298 K using 1 mM of LabMol-309 in D_2_O. The interaction between LabMol-309 and nsP4-CHIKV was studied using a Bruker Avance IIIHD 500 MHz in a solution of 20 mM (^2^H)_11_-Tris/HCL pH 7.5, 200 mM NaCl and 250 µM of LabMol-309. One-dimensional ^1^H spectra in the presence and absence of 3 µM NSP4-CHIKV were acquired, and the chemical shift difference was analyzed. The data processing and analysis were performed using TopSpin 4.09.

### The nsP4-CHIKV tridimensional model and structural analysis

The nsP4-CHIKV sequence (residues 1 to 611) was used to generate the 3D model by AlphaFold2, developed by DeepMind (https://alphafold.ebi.ac.uk/)^38^. The nsP4-CHIKV model was structurally refined for docking calculations at GalaxyRefine server^63^. Surface charge was calculated using APBS^64^ and residues conservation was analyzed with ConSurf, following the default parameters^65^. Pymol^66^ was used to render the 3D images.

### Molecular docking of nsP4-CHIKV and LabMol-309

The docking calculations were performed using the DockThor VS web^67,68^, focusing on the active binding site (Asp371 and Asp466 residues). The nsP4-CHIKV and LabMol-309 structures were prepared using the Protein Preparation Wizard^69^ and LigPrep tool^70^. The docking grid was centered at the active binding site; grid size 20 Å; and grid coordinates x, y and z of − 27.84 Å, 12.89 Å and 28.25 Å, respectively. The search algorithm precision mode was set up in the standard configuration of genetic algorithm parameters, with the soft docking mode activated. The PLIP server^71^ was used to analyze the protein–ligand patterns (hydrogen bonds, hydrophobic interaction, cation-π, π-stacking, water and salt bridge interactions). Poseview server^72,73^ was used to generate 2D interaction diagram and VMD program was used to render the 3D images^74^.

### Molecular dynamics simulations

The initial positions of the nsP4-CHIKV-bound LabMol-309 for the molecular dynamics (MD) simulations were obtained by the molecular docking results, and its topology parameterizations (Molid 814093) were obtained from the ATB server^75^. The MD simulations were performed using GROMACS package version 5.0.7^76^. The molecular system of the protein–ligand complex was modeled with the GROMOS54A7 force field^77^ and SPC water model^78^, using a cubic box solvated with 200 mM NaCl. The simulation was realized in ensemble NPT at 25 °C and 1.0 bar using a modified Berendsen thermostat with τ_T_ = 0.1 ps and Parrinello-Rahman barostat with τ_P_ = 2.0 ps and compressibility = 4.5 × 10^–5^·bar^–1^. A cutoff value of 12 Å was used for both Lennard–Jones, and Coulomb potentials and long-range electrostatic interactions were calculated using the Particle Mesh Ewald algorithm (PME)^79^. Energy minimization was performed with the steepest descent integrator and conjugate gradient algorithm, using 1000 kJ·mol^−1^·nm^−1^ as the maximum force criterion. One hundred thousand molecular dynamics steps were performed for each NVT and NPT equilibration, applying force constants of 1000 kJ·mol^−1^·nm^−2^ to all heavy atoms of the protein–ligand complex. At the end of preparation, a 100 ns MD simulation of the structural model of the protein–ligand complex was carried out for data acquisition. Next, the trajectory was aligned and analyzed according: RMSD of backbone atoms for protein and nonhydrogen atoms for the ligand, number of hydrogen bounds (cutoff distance of 3.5 Å and maximum angle of 30°) between protein and ligand, and number of contacts < 0.6 nm between all atoms of the protein and of the ligand.

### Cells and virus

BHK-21 cells were purchased from The Global Bioresource Center (ATCC) and maintained in Dulbecco's modified Eagle's medium (DMEM, Sigma-Aldrich) supplemented with 100U/mL of penicillin (Hyclone Laboratories), 100 mg/mL of streptomycin (Hyclone Laboratories), 1% dilution of stock of non-essential amino acids (Hyclone Laboratories) and 10% of fetal bovine serum (FBS, Hyclone Laboratories) in a humidified 5% CO2 incubator at 37 °C. BHK-21-Gluc-nSP-CHIKV-99659 cell line, harboring a replicative CHIKV replicon expressing *Gaussia* luciferase (Gluc) as a reporter gene, was maintained in DMEM 10% FBS with 500 µg/ml G418 (Sigma-Aldrich). The CHIKV replicon construct includes a T7 bacteriophage promotor followed by the viral 5' UTR region, the nsp1-4 coding sequence, the CHIKV subgenomic promoter (Sg) followed by the GLuc sequence and the expression cassette containing a ubiquitination sequence (Ubi) and the neomycin phosphotransferase gene (Neo-resistance gene), and the viral 3' UTR region. This construction and the development of this replicon cell line will be described elsewhere. The CHIKV expressing *nanoluciferase* reporter (CHIKV-*nanoluc*) used for the antiviral assays is based on the CHIKV isolate LR2006OPY1 (East/Central/South African genotype) and was produced, rescued, and titrated as previously described^40,41^.

### CHIKV replicon-based screenings

LabMol-309 at 200 mM in 100% DMSO was diluted with assay media to a final concentration of 1% (v/v) DMSO and was evaluated in a dose-dependent manner to determine its effectiveness (EC_50_) and cytotoxic (CC_50_) concentrations, as described in^80^. Approximately 2 × 10^4^ replicon cells/well in DMEM 10% FBS were seeded in a 96-well plate. After 16 h of incubation at 37 °C with 5% CO_2_, the medium was replaced with fresh DMEM supplemented with 2% FBS and compound was added to the cells at twofold serial dilutions. After a 48 h-incubation, 40 µL of the cells' supernatant containing secreted Gluc were mixed with 50 μl of *Renilla* luciferase Assay Reagent (Promega). The Gluc activity was measured using SpectraMax i3 Multi-mode Detection Platform (Molecular Devices). Replicon cells in 1% DMSO were used as negative control (0% inhibition). The compound concentration required to inhibit 50% of the Gluc activity (EC_50_) was estimated using the OriginPro 9.0 software. The cytotoxicity was evaluated through a cell proliferation-based MTT (3-(4,5-dimethylthiazol-2-yl)-2,5-diphenyltetrazolium bromide) assay, as described in^81^. The compound concentration required to cause 50% cytotoxicity (CC_50_) was estimated using the OriginPro 9.0 software. The dose–response curves were performed twice in duplicates. The EC_50_ and CC_50_ values were used to determine the compound's selectivity index (SI = CC_50_/EC_50_).

### Infection assays using CHIKV-nanoluc

To assess the antiviral activity of LabMol-309, BHK-21 cells were seeded at a density of 5 × 10^4^ cells per well into 48 well plates for 24 h. Then, cells were treated with LabMol-309 in a two-fold dilutions ranging from 10.78 to 100 µM in the presence or absence of CHIKV-*nanoluc* at a multiplicity of infection (MOI) of 0.1 PFU/cell. Samples were harvested using *Renilla*-luciferase lysis buffer (Promega®) 16 h post-infection (h.p.i.) and virus replication levels were quantified by measuring *nanoluciferase* activity using the *Renilla* luciferase Assay System (Promega®).

### Cell viability assays in BHK-21 cells

As previously described^40,41^, cell viability was measured by MTT [3-(4,5-dimethylthiazol-2-yl)-2,5-diphenyl tetrazolium bromide] assay (Sigma-Aldrich®). After this, the medium was replaced with the MTT solution at 1 mg/mL, cells were incubated for 30 min, after which the MTT solution was removed and replaced with 300 μL of DMSO (dimethyl sulfoxide) to solubilize the formazan crystals. The absorbance was measured at 490 nm on the Glomax microplate reader (Promega®). Cell viability was calculated according to the equation (T/C) × 100%, where T and C represent the mean optical density of the treated and untreated control groups, respectively. The values of CC_50_ and EC_50_ were used to calculate the selectivity index (SI = CC_50/_ EC_50_). The cytotoxic concentration of 50% (CC_50_) and the effective concentration of 50% inhibition (EC_50_) were calculated using GraphPad Prism 8.0.0 for Windows (GraphPad Software, San Diego, California USA, www.graphpad.com).

## Supplementary Information

Supplementary Information.

## Data Availability

The datasets generated and/or analysed during the current study are included in the are included in this published article [and its supplementary information files]. The raw data of all cellular assays presented in the manuscript were available in the Supplementary information. Additionally, the three-dimensional model of the protein generated using Alphafold is available upon request to the corresponding author.
