# Supplementary material for: Characterization of the RNA-dependent RNA polymerase from Chikungunya virus and discovery of a novel ligand as a potential drug candidate
Source: Sci Rep. 2022 Jun 22;12:10601. doi: 10.1038/s41598-022-14790-x (PMC9217121; doi:10.1038/s41598-022-14790-x)
Supplement: Supplementary file 1 — Supplementary Information. [file 41598_2022_14790_MOESM1_ESM.docx]

**SUPPLEMENTARY MATERIAL**

**Supplementary Material for**

**Characterization of the RNA-dependent RNA polymerase from Chikungunya virus and discovery of a novel ligand as a potential drug candidate**

Marjorie C. L. C. Freire^1^; Luis G. M. Basso^2^; Luis F. S. Mendes^3^; Nathalya C. M. R. Mesquita^1^; Melina Mottin^4^; Rafaela S. Fernandes^1^, Lucca R. Policastro^1^; Andre S. Godoy^1^; Igor A. Santos^5^, Uriel E. A. Ruiz^5^; Icaro P. Caruso^6,7^, Bruna K. P. Sousa^4^; Ana C. G. Jardim^5,6^, Fabio C. L. Almeida^7,8^; Laura H. V. G. Gil^9^; Carolina H. Andrade^4^; Glaucius Oliva^1,*^

^1^Institute of Physics of Sao Carlos, University of Sao Paulo, Sao Carlos, SP, Brazil.

^2^Physical Sciences Laboratory, State University of Northern Rio de Janeiro Darcy Ribeiro (UENF), Campos dos Goytacazes, RJ, Brazil.

^3^Departament of Physics, Ribeirao Preto School of Philosophy, Science and Literature, University of Sao Paulo, Ribeirao Preto, SP, Brazil.

^4^Laboratory for Molecular Modeling and Drug Design, Labmol, Faculty of Pharmacy, Universidade Federal de Goiás, Goiânia, GO, Brazil.

^5^Institute of Biomedical Sciences, Federal University of Uberlandia, Uberlandia, MG, Brazil.

^6^Institute of Biosciences, Humanities and Exact Sciences (Ibilce), Sao Paulo State University (Unesp), Campus Sao Jose do Rio Preto, Sao Jose do Rio Preto, SP, Brazil

^7^Institute of Medical Biochemistry (IBqM) Leopoldo de Meis, National Center of Nuclear Magnetic Resonance Jiri Jonas, Federal University of Rio de Janeiro, Rio de Janeiro, RJ, Brazil.

^8^National Center of Nuclear Magnetic Resonance (CNRMN), Center of Structural Biology and Bioimaging (CENABIO), Federal University of Rio de Janeiro, Rio de Janeiro, RJ, Brazil.

^9^Instituto Aggeu Magalhaes (IAM-FIOCRUZ), Recife, PE, Brazil.

**FIGURES**

**
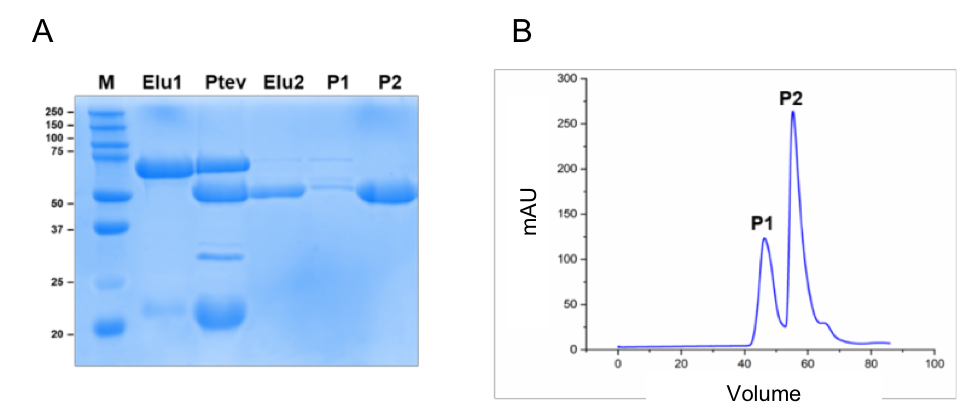
**

**Supplementary Figure 1. nsp4-CHIKV purification. A)** Acrylamide gel eletrophoresis of nsP4-CHIKV purification steps. M: molecular weight marker; Elu1: fraction eluted after the first affinity chromatography step; Ptev: fraction obtained after TEV protease cleavage; Elu2: fraction eluted after the second affinity chromatography step; P1 and P2: fractions eluted in gel filtration step. The P2 corresponds to the purified nsP4-CHIKV (54.54 kDa). **B)** Chromatogram of gel filtration step. in which the second peak (P2) corresponds to the purified nsP4-CHIKV (54.54 kDa).

**
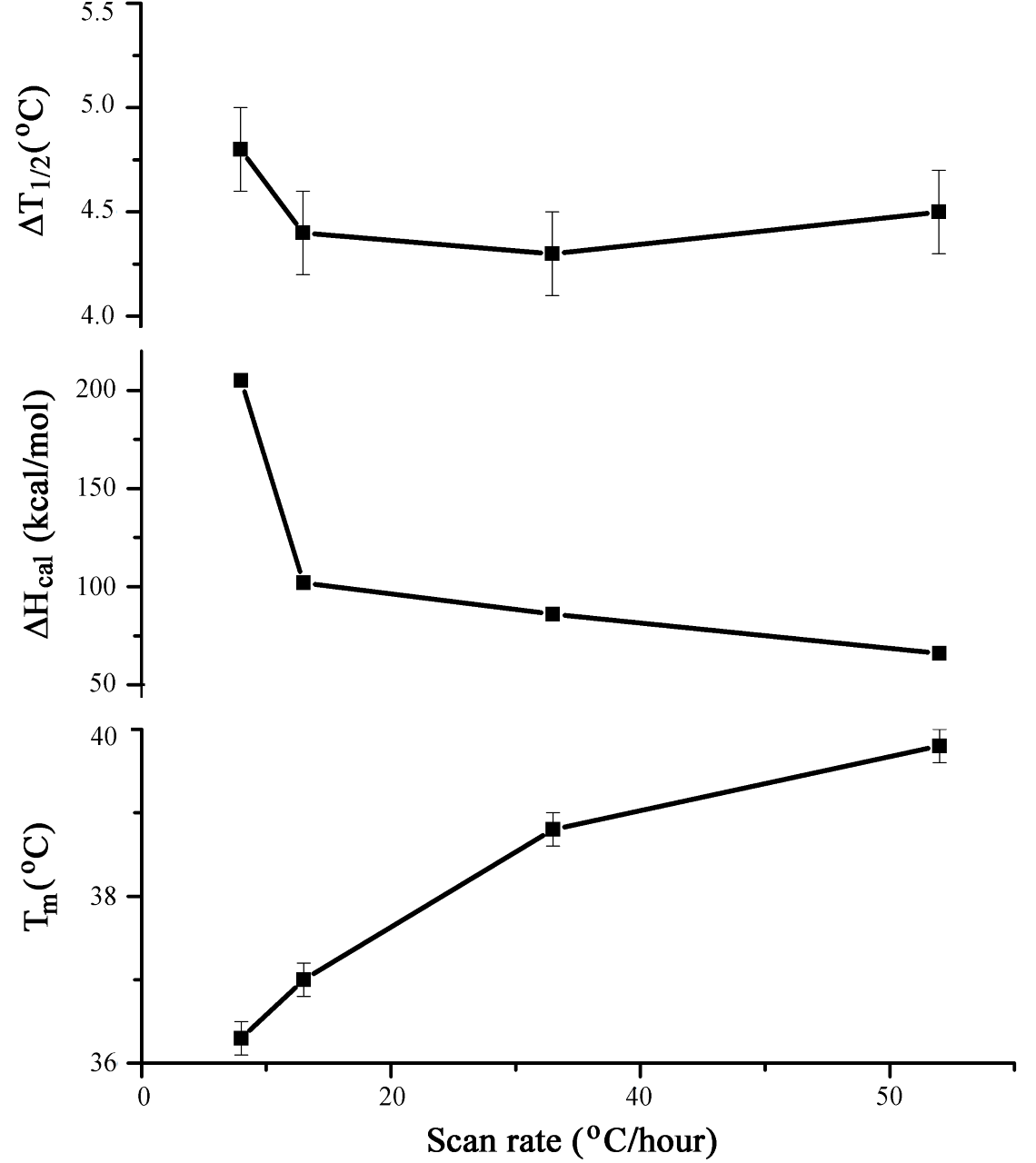
**

**Supplementary Figure 2.** **Dependences of the thermodynamic parameters (ΔH_cal_. T_m_ and ΔT_1/2_)on the heating rate.** This dependences are markedly non-linear. This feature illustrates the non-equilibrium character of its denaturation processes. The dependence on the heating scan might also suggest that the rates of the conformational transitions are rather low. compared with the heating rates.


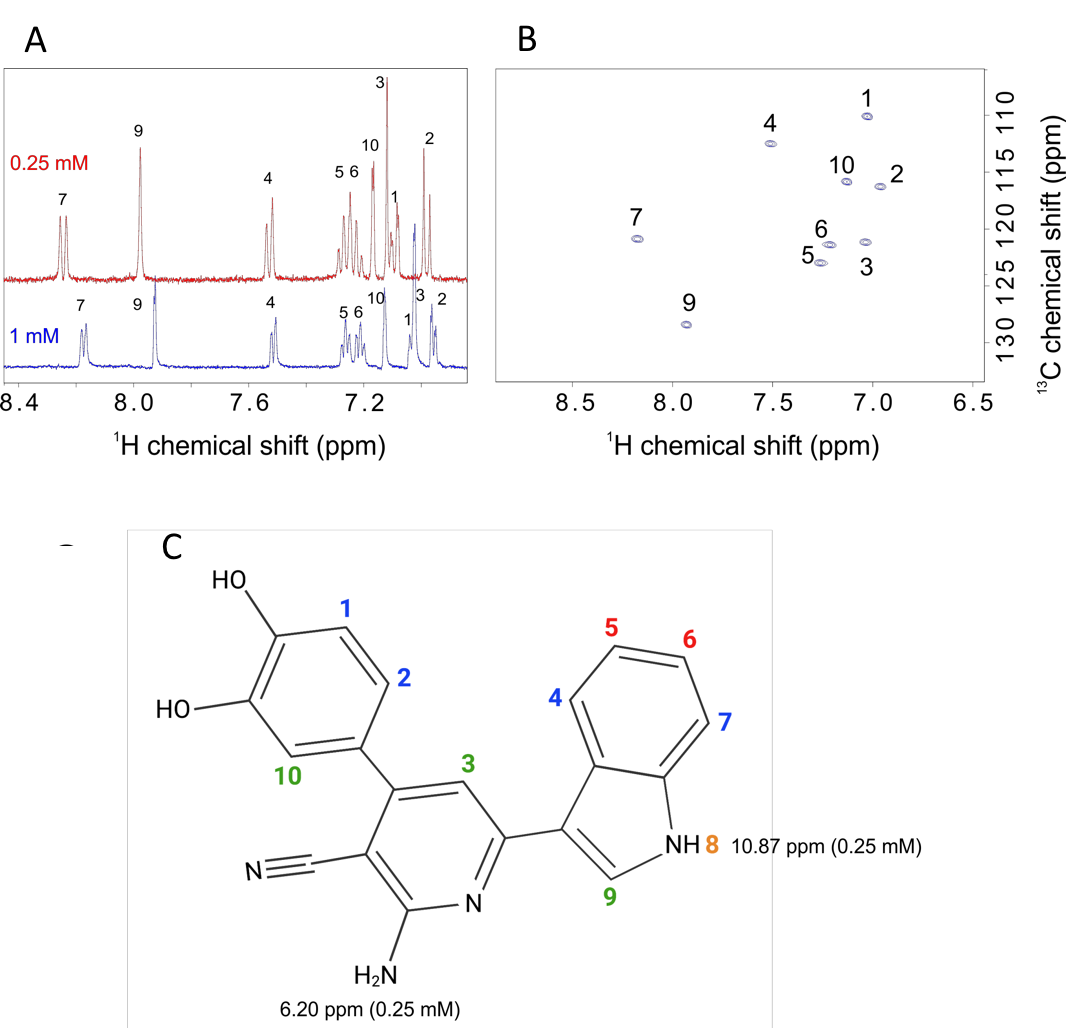


**Supplementary Figure 3.** LabMol-309 NMR assignment. (**A)** and (**B)** represents the NMR spectra of LabMol-309, and the respective positions of the protons resonances. The 1H-13C-HSQC, COSY and TOCSY were acquired at 298 K using 0.25 mM (red line) and 1 mM (blue line) of LabMol-309 in D2O. **C)** The positions of the protons in LabMol-309.

**TABLES**

**Supplementary Table 1. Methods used in the spectral deconvolution of nsP4-CHIKV using different protein spectral databases.** The secondary structural content for each method-base pair is presented in fraction of helix (H), sheets (S), turns (T), unordered structures (U) and the NRMSD.

| **Method** | **Database** | **H** | **S** | **T** | **U** | **NRMSD** |
| --- | --- | --- | --- | --- | --- | --- |
|  | Set 4 | 0.565 | 0.070 | 0.139 | 0.251 | 0.194 |
| SELCON3 | Set 7 | 0.583 | 0.059 | 0.146 | 0.254 | 0.198 |
|  | SP175 | 0.536 | 0.075 | 0.111 | 0.272 | 0.114 |
|  | **Average** | **0.561 ± 0.023** | **0.068 ± 0.008** | **0.132 ± 0.018** | **0.259 ± 0.011** |  |
|  |  |  |  |  |  |  |
|  | Set 4 | 0.552 | 0.053 | 0.149 | 0.245 | 0.037 |
| CONTIN | Set 7 | 0.559 | 0.047 | 0.147 | 0.248 | 0.037 |
|  | SP175 | 0.536 | 0.075 | 0.114 | 0.274 | 0.037 |
|  | **Average** | **0.549 ± 0.011** | **0.058 ± 0.014** | **0.136 ± 0.019** | **0.255 ± 0.015** |  |
|  |  |  |  |  |  |  |
|  | Set 4 | 0.610 | 0.070 | 0.100 | 0.220 | 0.012 |
| CDSSRT | Set 7 | 0.630 | 0.050 | 0.090 | 0.230 | 0.012 |
|  | SP175 | 0.560 | 0.070 | 0.100 | 0.270 | 0.009 |
|  | **Average** | **0.600 ± 0.036** | **0.063 ± 0.011** | **0.096 ± 0.005** | **0.240 ± 0.026** |  |
|  |  |  |  |  |  |  |

**Supplementary Table 2. Compounds tested against nsp4-CHIKV.** The 2D structure, name of compounds and the melting temperature (*T_m_*) obtained for each compound in DSF screening.

| **2D structure** | **Compound ID** | ***T_m_* (°C)** |  |
| --- | --- | --- | --- |
| 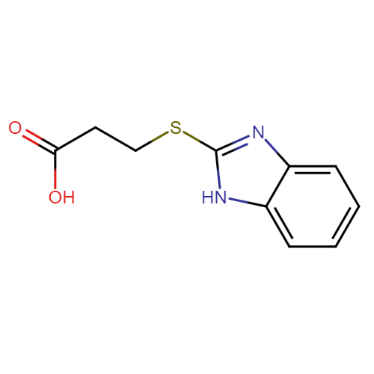 | LabMol-194 | 37.34 ± 0.14 |  |
| 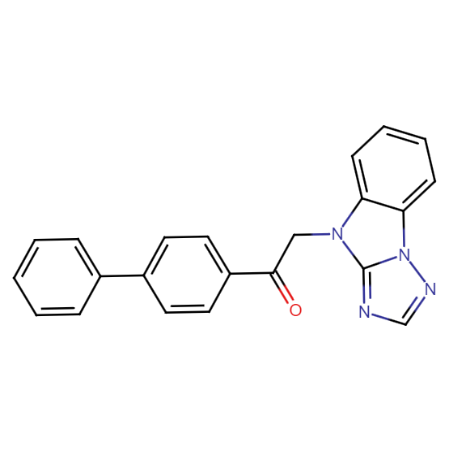 | LabMol-315 | 37.67 ± 1.05 |  |
| 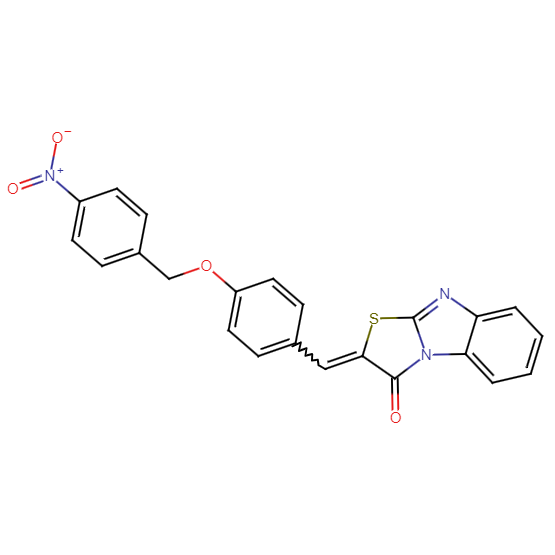 | LabMol-299 | 36.44 ± 0.53 |  |
| 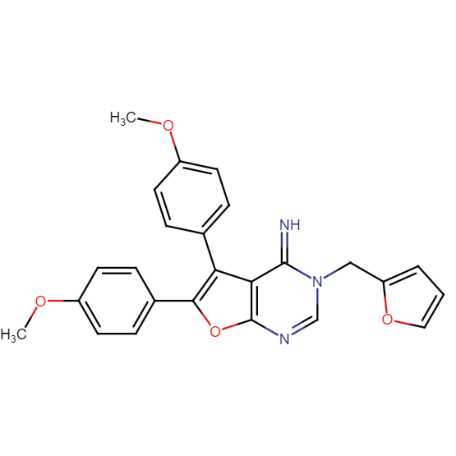 | LabMol-303 | 37.43 ± 0.92 |  |
| 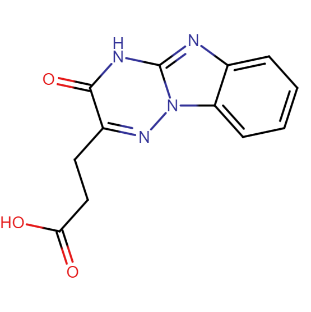 | LabMol-326 | 37.82 ± 0.65 |  |
| 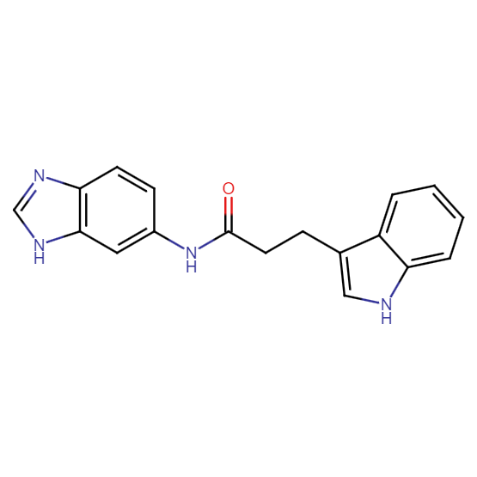 | LabMol-312 | 37.48 ± 0.36 |  |
| 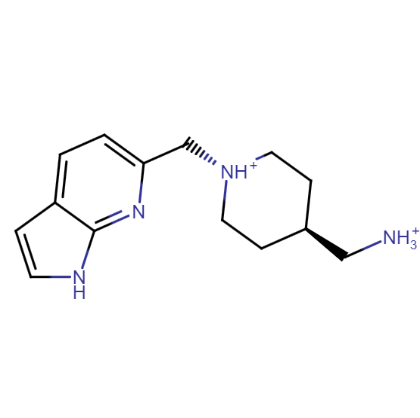 | LabMol-202 | 37.57 ± 0.45 |  |
| 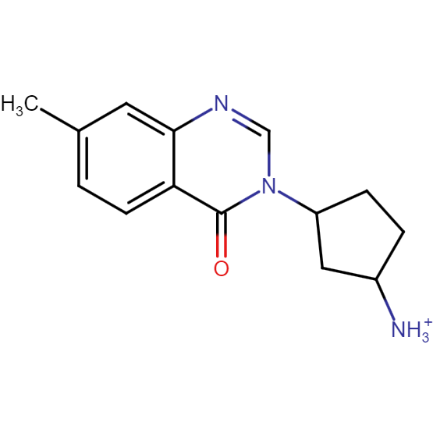 | LabMol-211 | 36.74 ± 0.26 |  |
| 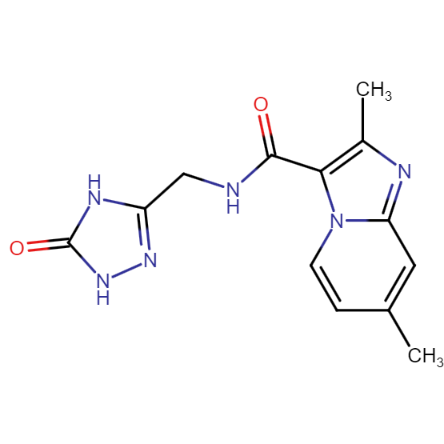 | LabMol-187 | 35.81 ± 0.19 |  |
| 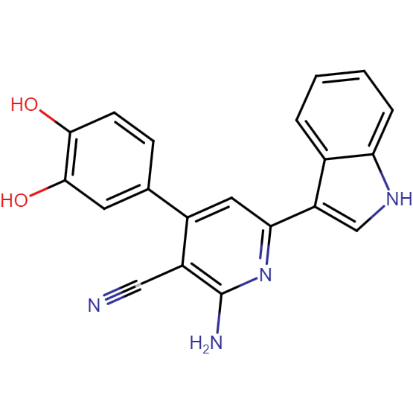 | LabMol-309 | 33.25 ± 0.37 |  |
| 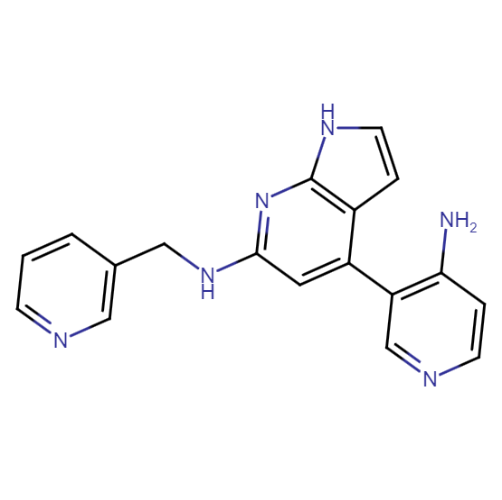 | LabMol-301 | 37.59 ± 0.14 |  |
| 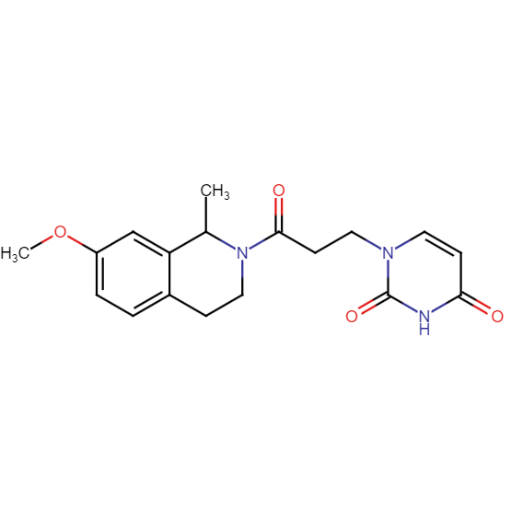 | LabMol-183 | 34.63 ± 0.96 | |

**Supplementary Table 3.** Percentage occupancy of hydrogen bonds formed between nsP4-CHIKV and LabMol-309 throughout the 100 ns MD simulations. The bold denotes significant percentage occupancy of hydrogen bonds higher than 5%.

| Donor | |  | Acceptor | | Percentage  Occupancy (%) |
| --- | --- | --- | --- | --- | --- |
| Residue | atom |  | Residue | atom |  |
| LYS295 | NZ |  | LABMOL-309 | N2 | 0.010 |
| LYS295 | NZ |  | LABMOL-309 | N3 | 0.030 |
| ASN468 | N |  | LABMOL-309 | N4 | 0.020 |
| LYS492 | NZ |  | LABMOL-309 | O1 | 0.160 |
| LYS492 | NZ |  | LABMOL-309 | O2 | 0.020 |
| LYS501 | NZ |  | LABMOL-309 | O1 | 1.070 |
| **LYS501** | **NZ** |  | **LABMOL-309** | **O2** | **19.158** |
| TYR504 | OH |  | LABMOL-309 | N1 | 0.080 |
| GLY507 | N |  | LABMOL-309 | N1 | 0.010 |
| LYS527 | NZ |  | LABMOL-309 | N1 | 0.010 |
| LYS527 | NZ |  | LABMOL-309 | N2 | 0.500 |
| LYS527 | NZ |  | LABMOL-309 | N3 | 1.020 |
| ARG573 | NE |  | LABMOL-309 | N1 | 1.380 |
| ARG573 | NH1 |  | LABMOL-309 | O1 | 0.030 |
| ARG573 | NH1 |  | LABMOL-309 | O2 | 3.620 |
| **ARG573** | **NH1** |  | **LABMOL-309** | **N1** | **5.659** |
| ARG573 | NH1 |  | LABMOL-309 | N2 | 0.180 |
| ARG573 | NH2 |  | LABMOL-309 | O1 | 0.040 |
| **ARG573** | **NH2** |  | **LABMOL-309** | **O2** | **7.219** |
| **ARG573** | **NH2** |  | **LABMOL-309** | **N1** | **7.019** |
| ARG573 | NH2 |  | LABMOL-309 | N2 | 0.330 |
| ARG573 | NH2 |  | LABMOL-309 | N3 | 0.010 |
| TYR574 | OH |  | LABMOL-309 | N1 | 0.080 |
| **LABMOL-309** | **O1** |  | **ASP371** | **OD1** | **11.049** |
| **LABMOL-309** | **O1** |  | **ASP371** | **OD2** | **88.341** |
| **LABMOL-309** | **O2** |  | **GLU369** | **OE1** | **73.973** |
| **LABMOL-309** | **O2** |  | **GLU369** | **OE2** | **27.697** |
| LABMOL-309 | N2 |  | ASP467 | OD2 | 0.010 |
| LABMOL-309 | N2 |  | TYR504 | OH | 0.200 |
| LABMOL-309 | N2 |  | TYR574 | OH | 0.890 |
| LABMOL-309 | N4 |  | THR370 | OG1 | 0.030 |
| LABMOL-309 | N4 |  | ASP466 | OD1 | 1.120 |
| LABMOL-309 | N4 |  | ASP466 | OD2 | 0.460 |
| **LABMOL-309** | **N4** |  | **ASP466** | **O** | **5.489** |
| **LABMOL-309** | **N4** |  | **ASP467** | **OD1** | **43.086** |
| **LABMOL-309** | **N4** |  | **ASP467** | **OD2** | **15.968** |
| **LABMOL-309** | **N4** |  | **ASN468** | **OD1** | **9.699** |
| LABMOL-309 | N4 |  | ASN468 | ND2 | 0.020 |
| LABMOL-309 | N4 |  | ASN468 | O | 0.010 |

**Supplementary Table 4.** Raw data of Replicon-based assays – EC_50_.

**EC_50_**

| **RepCHIKV (RLUs)** |  |  |
| --- | --- | --- |
|  | **Experiment 1** | **Experiment 2** |
| **Replicate 1 (R1)** | 10624 | 10503 |
| **Replicate 2 (R2)** | 10638 | 12920 |
| **Average** | 10631 | 11711.5 |

| **LabMol-309** |  |  |  |  |  |  |  |  |  |  |
| --- | --- | --- | --- | --- | --- | --- | --- | --- | --- | --- |
| **Experiment 1** |  |  |  |  |  |  |  |  |  |  |
| **[uM]** | **20** | **10** | **5** | **2.5** | **1.25** | **0.625** | **0.3125** | **0.15625** | **0.078125** | **0.039063** |
| **R1** | 2362 | 7263 | 11043 | 12380 | 12366 | 11812 | 12838 | 10328 | 10314 | 8154 |
| **R2** | 2552 | 5589 | 9598 | 10408 | 10300 | 10570 | 6885 | 7736 | 8667 | 8600 |
| **Average** | 2457 | 6426 | 10320.5 | 11394 | 11333 | 11191 | 9861.5 | 9032 | 9490.5 | 8377 |
| **Gluc activity (%)** | 23.11165 | 60.44586586 | 97.0793 | 107.1771235 | 106.6033 | 105.2676 | 92.76173 | 84.95908 | 89.27194 | 78.79786 |
| **STDEV (%)** | 1.26376 | 11.13438766 | 9.611225 | 13.1164949 | 13.74172 | 8.260997 | 39.59559 | 17.24034 | 10.9548 | 2.966509 |

| **LabMol-309** |  |  |  |  |  |  |  |  |  |  |
| --- | --- | --- | --- | --- | --- | --- | --- | --- | --- | --- |
| **Experiment 2** |  |  |  |  |  |  |  |  |  |  |
| **[uM]** | **20** | **10** | **5** | **2.5** | **1.25** | **0.625** | **0.3125** | **0.15625** | **0.078125** | **0.039063** |
| **R1** | 1782 | 6602 | 10192 | 10800 | 10166 | 11326 | 8222 | 8559 | 8708 | 9922 |
| **R2** | 2808 | 6008 | 9896 | 8842 | 7844 | 9288 | 7276 | 7425 | 9099 | 7250 |
| **Average** | 2295 | 6305 | 10044 | 9821 | 9005 | 10307 | 7749 | 7992 | 8903.5 | 8586 |
| **Gluc activity (%)** | 19.59612 | 53.83597319 | 85.76186 | 83.85774666 | 76.89024 | 88.00751 | 66.16573 | 68.24062 | 76.02357 | 73.31256 |
| **STDEV (%)** | 6.194694 | 3.58640164 | 1.787163 | 11.82184244 | 14.01957 | 12.30486 | 5.711677 | 6.846767 | 2.360746 | 16.13277 |

**Supplementary Table 5.** Raw data of Replicon-based assays – CC_50_.

**CC_50_**

| **RepCHIKV (absorbance at 570 nm)** |  |  |
| --- | --- | --- |
|  | **Experiment 1** | **Experiment 2** |
| **Replicate 1 (R1)** | 0.3839 | 0.3297 |
| **Replicate 2 (R2)** | 0.4178 | 0.4326 |
| **Average** | 0.40085 | 0.38115 |

| **LabMol-309** |  |  |  |  |  |  |  |  |  |  |
| --- | --- | --- | --- | --- | --- | --- | --- | --- | --- | --- |
| **Experiment 1** |  |  |  |  |  |  |  |  |  |  |
| **[uM]** | **20** | **10** | **5** | **2.5** | **1.25** | **0.625** | **0.3125** | **0.15625** | **0.078125** | **0.039063** |
| **R1** | 0.097 | 0.0848 | 0.1207 | 0.2967 | 0.3456 | 0.418 | 0.071 | 0.4756 | 0.4339 | 0.3901 |
| **R2** | 0.0912 | 0.0868 | 0.1385 | 0.294 | 0.4327 | 0.5291 | 0.473 | 0.5982 | 0.5873 | 0.6346 |
| **Average** | 0.0941 | 0.0858 | 0.1296 | 0.29535 | 0.38915 | 0.47355 | 0.272 | 0.5369 | 0.5106 | 0.51235 |
| **Cell viability (%)** | 23.47512 | 21.40452 | 32.3313 | 73.68093 | 97.0812 | 118.1365 | 67.85581 | 133.9404 | 127.3793 | 127.8159 |
| **STDEV (%)** | 1.023131 | 0.352804 | 3.139953 | 0.476285 | 15.3646 | 19.59824 | 70.91354 | 21.62687 | 27.06004 | 43.13025 |

| **LabMol-309** |  |  |  |  |  |  |  |  |  |  |
| --- | --- | --- | --- | --- | --- | --- | --- | --- | --- | --- |
| **Experiment 2** |  |  |  |  |  |  |  |  |  |  |
| **[uM]** | **20** | **10** | **5** | **2.5** | **1.25** | **0.625** | **0.3125** | **0.15625** | **0.078125** | **0.039063** |
| **R1** | 0.0912 | 0.0811 | 0.1303 | 0.2574 | 0.4427 | 0.5065 | 0.0854 | 0.4485 | 0.4944 | 0.5443 |
| **R2** | 0.1043 | 0.0825 | 0.1269 | 0.3233 | 0.4444 | 0.6005 | 0.4581 | 0.4695 | 0.473 | 0.5192 |
| **Average** | 0.09775 | 0.0818 | 0.1286 | 0.29035 | 0.44355 | 0.5535 | 0.27175 | 0.459 | 0.4837 | 0.53175 |
| **Cell viability (%)** | 25.64607 | 21.46137 | 33.74 | 76.17736 | 116.3715 | 145.2184 | 71.29739 | 120.425 | 126.9054 | 139.512 |
| **STDEV (%)** | 2.430303 | 0.259727 | 0.630766 | 12.22572 | 0.315383 | 17.43881 | 69.14304 | 3.895905 | 3.970113 | 4.656534 |

**Supplementary Table 6.** **Raw data from cell viability and CHIKV-*nanoluc* replication rate.**

| **LabMol-309**  **Concentration** | **Cells Viability Raw data** | | | | | | **CHIKV Replication Rate Raw data** | | | | | |
| --- | --- | --- | --- | --- | --- | --- | --- | --- | --- | --- | --- | --- |
|  | **Experiment 1** | | | **Experiment 2** | | | **Experiment 1** | | | **Experiment 2** | | |
| 100 μM | 0.16268141 | 0.15940569 | 0.19535184 | 0.43464547 | 0.52458866 | 0.40546728 | 50 | 175 | 110 | 510 | 1015 | 2325 |
| 50 μM | 0.18181899 | 0.32228734 | 0.31792173 | 0.49031271 | 0.54311497 | 0.54362338 | 195 | 125 | 95 | 40040 | 50820 | 76010 |
| 25 μM | 0.39356699 | 0.37946687 | 0.34952676 | 0.62278193 | 0.68927388 | 0.584814 | 860 | 795 | 2720 | 2308000 | 2753000 | 4959000 |
| 12.5 μM | 0.45515081 | 0.36157638 | 0.43924437 | 0.72638563 | 0.86537469 | 0.79827139 | 12130 | 28920 | 25430 | 28840000 | 23910000 | 29160000 |
| 6.25 μM | 0.42176537 | 0.36623583 | 0.4754789 | 0.73916343 | 0.84530342 | 0.9647667 | 95000 | 89740 | 64440 | 30500000 | 28970000 | 26810000 |
| 3.12 μM | 0.50575559 | 0.40097701 | 0.48154542 | 0.9268913 | 0.80727913 | 0.98713326 | 173800 | 150200 | 125700 | 48280000 | 24420000 | 23290000 |
| 1.56 μM | 0.49075037 | 0.38405706 | 0.53842885 | 0.86831927 | 1.05132904 | 1.00295509 | 175100 | 162500 | 217000 | 71800000 | 53920000 | 53110000 |
| 0.78 μM | 0.46359433 | 0.53201885 | 0.71865811 | 1.00258903 | 1.09950888 | 1.16943187 | 232800 | 162200 | 199100 | 53080000 | 81470000 | 61330000 |

**Supplementary Table 7: Normalized data of cell viability in BHK-21 cells.**

| **LabMol-309**  **Concentration** | **Cells Viability (%)** | | | | | |
| --- | --- | --- | --- | --- | --- | --- |
|  | **Experiment 1** | | | **Experiment 2** | | |
| 100 μM | 28.5405976 | 27.9659103 | 34.2722528 | 43.4645473 | 52.4588656 | 40.5467278 |
| 50 μM | 31.8980685 | 56.5416387 | 55.7757414 | 49.0312707 | 54.3114971 | 54.362338 |
| 25 μM | 69.0468396 | 66.5731345 | 61.320484 | 62.2781926 | 68.9273876 | 58.4813997 |
| 12.5 μM | 79.8510192 | 63.4344531 | 77.0604166 | 72.6385633 | 86.5374694 | 79.8271388 |
| 6.25 μM | 73.9939247 | 64.2519003 | 83.4173515 | 73.9163434 | 84.5303416 | 96.4766701 |
| 3.12 μM | 88.7290517 | 70.3468438 | 84.4816521 | 92.6891297 | 80.7279131 | 98.7133264 |
| 1.56 μM | 86.0965557 | 67.3784319 | 94.4612023 | 86.831927 | 105.132904 | 100.295509 |
| 0.78 μM | 81.3323386 | 93.3366411 | 126.080371 | 100.258903 | 109.950888 | 116.943187 |

**Supplementary Table 8: Normalized data of CHIKV-*nanoluc* replication rate in BHK-21 cells.**

| **LabMol-309**  **Concentration** | **CHIKV Replication Rate (%)** | | | | | |
| --- | --- | --- | --- | --- | --- | --- |
|  | **Experiment 1** | | | **Experiment 2** | | |
| 100 μM | 0.02524828 | 0.08836896 | 0.05554621 | 0.00078109 | 0.001554523 | 0.003560854 |
| 50 μM | 0.09846827 | 0.06312069 | 0.04797172 | 0.061323259 | 0.077833367 | 0.11641311 |
| 25 μM | 0.43427033 | 0.40144757 | 1.37350617 | 3.534817235 | 4.216356953 | 7.594956096 |
| 12.5 μM | 6.12523155 | 14.6036023 | 12.8412727 | 44.16989994 | 36.61935879 | 44.65999592 |
| 6.25 μM | 47.9717227 | 45.3156042 | 32.539977 | 46.71227282 | 44.36900143 | 41.06085359 |
| 3.12 μM | 87.7630043 | 75.8458185 | 63.4741637 | 73.94323055 | 37.40044926 | 35.66979784 |
| 1.56 μM | 88.4194595 | 82.0568942 | 109.577514 | 109.9652849 | 82.58117215 | 81.34061671 |
| 0.78 μM | 117.555969 | 81.9054045 | 100.538632 | 81.29467021 | 124.7753727 | 93.92995712 |
